# The Pnictogen Bond: The Covalently Bound Arsenic Atom in Molecular Entities in Crystals as a Pnictogen Bond Donor

**DOI:** 10.3390/molecules27113421

**Published:** 2022-05-25

**Authors:** Arpita Varadwaj, Pradeep R. Varadwaj, Helder M. Marques, Koichi Yamashita

**Affiliations:** 1 Department of Chemical System Engineering, School of Engineering, The University of Tokyo 7-3-1, Tokyo 113-8656, Japan; yamasita@chemsys.t.u-tokyo.ac.jp; 2 Molecular Sciences Institute, School of Chemistry, University of the Witwatersrand, Johannesburg 2050, South Africa; helder.marques@wits.ac.za

**Keywords:** pnictogen bonding, arsenic as pnictogen bond donor, inter- and intra-molecular geometry, directionality, crystal structure analysis, ICSD and CSD database analyses, MESP model description, sum of the van der Waals radii concept, pro-molecular charge-density based IGM-δg analysis

## Abstract

In chemical systems, the arsenic-centered pnictogen bond, or simply the arsenic bond, occurs when there is evidence of a net attractive interaction between the electrophilic region associated with a covalently or coordinately bound arsenic atom in a molecular entity and a nucleophile in another or the same molecular entity. It is the third member of the family of pnictogen bonds formed by the third atom of the pnictogen family, Group 15 of the periodic table, and is an inter- or intramolecular noncovalent interaction. In this overview, we present several illustrative crystal structures deposited into the Cambridge Structure Database (CSD) and the Inorganic Chemistry Structural Database (ICSD) during the last and current centuries to demonstrate that the arsenic atom in molecular entities has a significant ability to act as an electrophilic agent to make an attractive engagement with nucleophiles when in close vicinity, thereby forming σ-hole or π-hole interactions, and hence driving (in part, at least) the overall stability of the system’s crystalline phase. This overview does not include results from theoretical simulations reported by others as none of them address the signatory details of As-centered pnictogen bonds. Rather, we aimed at highlighting the interaction modes of arsenic-centered σ- and π-holes in the rationale design of crystal lattices to demonstrate that such interactions are abundant in crystalline materials, but care has to be taken to identify them as is usually done with the much more widely known noncovalent interactions in chemical systems, halogen bonding and hydrogen bonding. We also demonstrate that As-centered pnictogen bonds are usually accompanied by other primary and secondary interactions, which reinforce their occurrence and strength in most of the crystal structures illustrated. A statistical analysis of structures deposited into the CSD was performed for each interaction type As···D (D = N, O, S, Se, Te, F, Cl, Br, I, arene’s π system), thus providing insight into the typical nature of As···D interaction distances and ∠R–As···D bond angles of these interactions in crystals, where R is the remainder of the molecular entity.

## 1. Introduction

Pnictogen bonding, unlike other noncovalent interactions, is among the least theoretically and computationally studied chemical interactions [1,2,3,4,5,6], yet has been featured in applications in many areas such as catalysis [7,8,9,10], coordination chemistry [3,11,12,13,14,15], photovoltaics [16,17], and supramolecular chemistry [4,5]. There are relatively few reviews [3,18,19,20,21,22], original papers (for example [1,5,23,24,25,26,27,28,29]), overviews [30], and comments on the strength [31], nature [32,33], and propensity of elements of the pnictogen family (Group 15) to engage in pnictogen bonding [34,35,36]. The reason for this is probably because recent work on noncovalent interactions has focused largely on exploring σ-hole and π-hole interactions [37,38,39,40,41,42,43,44,45,46,47,48] associated with the main elements of the periodic table, with particular attention to the elements of Groups 14, 16, and 17, including hydrogen bonding [49].

A σ-hole on an atom A generally appears on its electrostatic surface along the extension of the R–A covalent bond and is deficient in electron density compared to the lateral sites of A; R is the remainder of the molecular entity [37,50,51]. A π-hole is generally observed on the electrostatic surface of a delocalized electron system, which may also be electron density deficient and, for example, appears on the multiple bonds in HCCH, P_2_, N_2_, and O_2_, and on the centroid regions of arene moieties [42,52], as well as on the surface of N and P in NO_3_^-^ [53]/R-NO_2_ [54] and PO_2_X (X = F, Cl) [55], respectively. Studies on noncovalent interactions formed by Group 15 elements are possibly limited because it is usually assumed that the elements of this group, such as N, are electronegative, resist polarization, and are therefore often negative. Another possible reason is that attention is still focused on understanding the modes of inter- and intramolecular interactions formed by hydrogen [56,57] and halogen [58,59,60,61,62,63,64,65] in molecular entities, even though they are governed by the same mechanism [66]. A fundamental understanding of the reaction behavior of halogen derivatives in the solid state that are important for the in-silico design of functional materials is one of the key concerns of many research groups, since elements of Group 17 in molecular entities have been featured in a significant number of engineered, functional, crystalline materials.

What, then, is a pnictogen bond and why is it important? The pnictogen bond in chemical systems occurs when there is evidence of a net attractive interaction between the electrophilic region associated with a covalently or coordinately bound pnictogen atom in a molecular entity and a nucleophile in another or the same molecular entity. It is formed by the atoms of the pnictogen family, Group 15 of the periodic table, and is an inter- or intramolecular noncovalent interaction. The nitrogen bond, the phosphorous bond, the arsenic bond, the stibium bond, and the bismuth bond are all manifestations of the pnictogen bond. A pnictogen bond features geometric and electronic characteristics akin to those of the hydrogen bond, triel bond (Group 13), tetrel bond (Group 14), chalcogen bond (Group 16), halogen bond (Group 17), arogen bond (Group 18), and other noncovalent interactions, and is therefore regarded as a sister interaction. The importance of pnictogen bonds emerges from its implication in the design and discovery of several functional materials [16,17], such as organic-inorganic halide perovskite semiconductors and in many other areas of materials science [3,4,5,6,7,8,9,10,11,12,13,14,15].

Some studies [5,30,67,68] have already provided illustrative overviews of pnictogen bonding formed by the first three elements of Group 15, N, P, and As. However, accounts elucidating the basis of such noncovalent interactions involving the heavier pnictogen derivatives, As, Sb, Bi, and (theoretically) Mc, are rare [5,30,67,68]. This is clearly a gap in our knowledge since the typical inter- and intramolecular geometry, orientation, energy, and charge density profiles of arsenic-, antimony-, or bismuth-centered noncovalent interactions have rarely been examined computationally, although a few studies have discussed the characteristics of arsenic-centered noncovalent interactions and their technical implications in materials design and discovery [69,70]; we also have recently provided our viewpoint on the diversity of stibium bonding in chemical systems. The relatively few reports available to date have not yet provided any rigorous basis for an in-depth understanding of the bonding topologies of covalently bound pnictogen atoms. We anticipate that many studies focusing on these features will appear in future reports that use computational and theoretical tools, as well as synthesis, to capture how and to what extent a heavier pnictogen derivative in a molecular entity would react when in proximity to a variety of electron density donors. To this end, more comprehensive studies are essential to fully elucidate the circumstances under which these noncovalent interactions occur in chemical systems. This was the case with hydrogen bonding, halogen bonding and chalcogen bonding; in fact, several thousands of studies eventually led to some appreciation—and formal definition—of these interactions [49,71,72]. It is noteworthy that over fifty definitions had been proposed for hydrogen bonding over many decades before a revised definition was adopted in 2011 [49]. Therefore, the noncovalent chemistry aspects of each member of the pnictogen family deserves attention and individual exploration since they are yet to be only partially understood.

Our search of the Inorganic Chemistry Structure Database (ICSD) [73,74] and the Cambridge Structural Database (CSD) [75,76] has shown that the proficiency of the later elements of the pnictogen family in forming pnictogen-bonding interactions is remarkably high. In this overview, we, therefore, have focused attention on several crystal structures, containing arsenic, deposited into the two databases, and on where the stability of the crystal lattice appears to be driven (at least in part) by arsenic-centered pnictogen bonding (or simply, by arsenic bonds). We show that arsenic in molecular entities can display positive σ-holes that have a significant ability to engage attractively with sites of differing electrostatic potential (generally negative) on the same or different molecular entities, to form intra- and/or intramolecular interactions of various kinds (such as σ-hole···lone pair and σ-hole···π). We emphasize that our sampling of the databases is not comprehensive; rather, we have chosen illustrative, representative, chemical systems containing arsenic, and have performed molecular electrostatic surface potential (MESP) analyses to demonstrate the way specific regions on the electrostatic surfaces of these molecular entities are in attractive engagement with interacting partners, aiding in the overall stability of the crystal lattice. Although the presence of arsenic-centered pnictogen-bonding interactions in the crystals primarily emerged from inspection of the inter- or intramolecular distances, and their directional features, validating the “less than the sum of the van der Waals radii” concept [77,78,79], application of the MESP model to some molecular entities enabled us to confirm the feasibility of coulombic interactions between interacting partner entities of the crystal lattices. In addition, we have also applied the charge-density based pro-molecular independent gradient model (IGM) [80,81] approach wherever we thought necessary, to some selected systems, to illustrate and confirm the presence of interactions in the crystalline systems we examined.

We note further that while this work provides an overview of the occurrence and likelihood of As-centered noncovalent interactions, our characterization may have missed or exaggerated their presence, since we did not perform a robust computational analysis of crystals, or isolated molecular complexes, to substantiate their identification and subsequent characterization. One of our primary concerns is to show the reader that As-centered pnictogen bonding in crystals does not appear alone, but appears along with its sister interactions: hydrogen bonds, chalcogen bonds, halogen bonds, and/or other noncovalent bonds. These may act either as the cause, or as a consequence, of pnictogen bonding; we show this when discussing their occurrence in the chosen illustrative crystal systems.

Statistical analysis was also performed on the intermolecular bond distances and bond angles, As···D (D = N, O, S, Se, Te, F, Cl, Br, I, arene’s π) and ∠R–As···D, respectively, obtained from CSD searches of the crystal structures. This was done to glean insight into the range of intermolecular distances and angles where As-centered noncovalent bonds can occur. However, the statistical data may not be completely reliable, given the appreciable number of outliers that emerged when the intermolecular geometry used as a constraint was made more flexible.

## 2. The “Less Than the Sum of the Van der Waals Radii” Criterion

According to Alvarez [82], the van der Waals radii of atoms and their associated van der Waals surfaces are extensively used for crystal packing and supramolecular interaction analysis. This is nontrivial since they describe the space-filling dimensions of atoms in molecular entities, and the 0.001 a.u. contour of a molecular entity’s charge density typically lies beyond the van der Waals radii of its component atoms [83]. In crystallography, the concept of “less than the van der Waals radii of the bonded atomic basins” has been widely applied to determine whether two atoms A and B on the same or two different molecular entities are bonded to each other [78,84,85] because it allows for a rationalization of whether the two atoms in close proximity overlap each other. The overlapping is readily appreciated when the interatomic distance between A and B is less than the sum of their van der Waals (vdW) radii. Traditionally, this concept has been used as a criterion for accepting or rejecting inter- and intramolecular interactions in a wide variety of chemical systems, regardless of whether the stability of the chemical system emerges from first-principles calculations or X-ray and neutron diffraction measurements. However, it is well-known that a rigid adherence to this criterion is a pitfall in the search for chemical interactions [78] because it may exclude genuine noncovalent interactions. Critical questions posed include: Are the proposed van der Waals radii of atoms precisely known? Is it necessarily the case that the vdW radius of an atom in one molecular entity is precisely the same in another different molecular entity? For example, is the vdW radius of O in H_2_O precisely the same as in HCOOH? Should the vdW radius of an atom be transferable from one system to the other?

The answer to all four questions is clearly “no”. The vdW radius of an atom is not a precise measure and indeed different authors have proposed different values of vdW radii for any given atom. For example, the vdW radius of Sb has been reported as 2.20 [86], 2.06 [87], and 2.47 Å [82], displaying very different radii values. This variation may not be expected since the uncertainty in the vdW radius of an atom could be as large as ±0.2 Å [77]. Therefore, it is very likely that the vdW radii of atoms vary from system to system and that the A···B distance can exceed the vdW radii sum by several tenths of an Ångstrom [77,88]. Furthermore, it should be kept in mind that the determination of vdW radii was based on a hard sphere model that presumes spherical symmetry. In reality, however, the charge density around atoms in a molecular entity is anisotropic, and this anisotropy varies from one molecular entity to another, depending on the bonding environment in which the atom finds itself. Despite considering these serious issues, the same vdW radius of a given atom has often been assumed to be transferable from one system to another, calling into question the (mis)use of the concept of “less than the sum of the vdW radii”. A stringent adherence to the criterion will undoubtedly result in a significant number of close contacts, but also reject numerous genuine interactions in molecular and crystal entities. In what follows, we will show that, on several occasions, the intermolecular bond distances associated with arsenic bonds in various crystals can be longer than the sum of the vdW radii of the interacting atomic basins. Furthermore, we emphasize that the vdW radii of the atoms are not unique values and that they vary from system to system. At best, the concept of “the sum of vdW radii” is a useful guide, but a guide that has to be treated with circumspection.

## 3. Directionality and Bonding Characterization

**Scheme 1:**
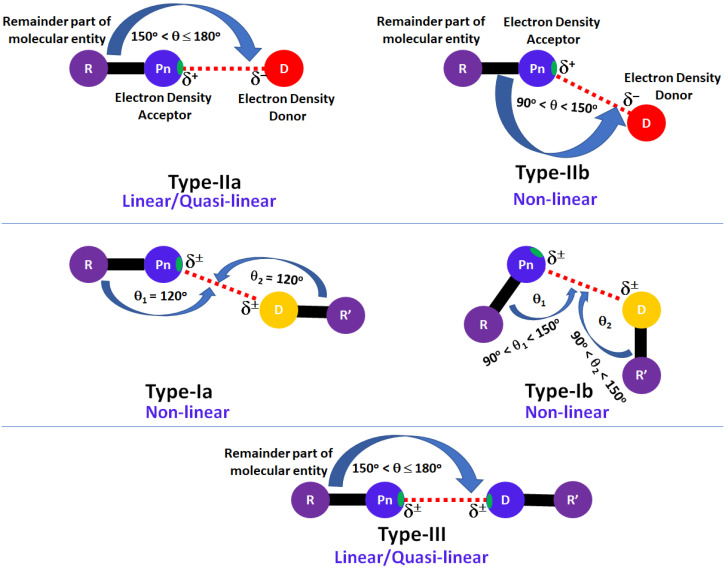
Geometric topology of chemical bonding interactions formed by the covalently bonded pnictogen atom in molecular complexes and crystals, where Pn, D, R, and R’ represent the pnictogen atom, the donor fragment, and the remainder of the molecular entities associated with Pn and D, respectively. The green region on covalently bonded atom Pn (see top panel, for example) signifies a positive σ-hole.

The concepts of Type-I, Type-II, and Type-III geometric topologies of bonding (Scheme 1) [38,89] are utilized in this overview to recognize and characterize arsenic-centered pnictogen bonds in the chosen crystal structures. This classification relies on the directionality between interacting atomic basins Pn and D, and the nature of the charge polarity (*δ*^±^) centered on specific regions on their surfaces. We recognize that Type-IIa and Type-IIb topologies of bonding occur between interacting molecular entities when 150° < *θ* < 180° and 90° < *θ* < 140°, respectively, where *θ* is the angle, *θ* = ∠R-Pn···D, of approach of the electrophile on Pn and D is the electron density donor (Scheme 1). The first are either linear, or quasi-linear, whereas the second are nonlinear and often accompanied by other (secondary) interactions. This means that Type-IIa interactions are directional, whereas Type-IIb are bent and nonlinear. However, the surface regions on the interacting atomic domains Pn and D have opposite charge polarity and the interacting surface portion of Pn is positive in both cases. 

Type-I topologies generally show up in two different flavors and are nonlinear; the *trans* form of Type-I bonding (Type-Ia) between interacting domains occurs when the angles of interaction are such that *θ*_1_ ≈ *θ*_2_ ≈ 120°; the *cis* form of Type-I bonding (Type-Ib) between interacting domains occurs when the angles of interaction are such that 90° < *θ*_1_ ≠ *θ*_2_ ≠ 120° < 150°. While both interacting atoms bonded to each other via a Type-I topology are generally caused by negative lateral regions on the interacting atoms, they can both be positive in adducts. 

A Type-III bonding topology between interacting atoms occurs when both interacting domains Pn and D have the same surface charge polarity, but of same or different magnitudes, and the angle of interaction between them is similar to that of a Type-IIa interaction.

**Scheme 2:**
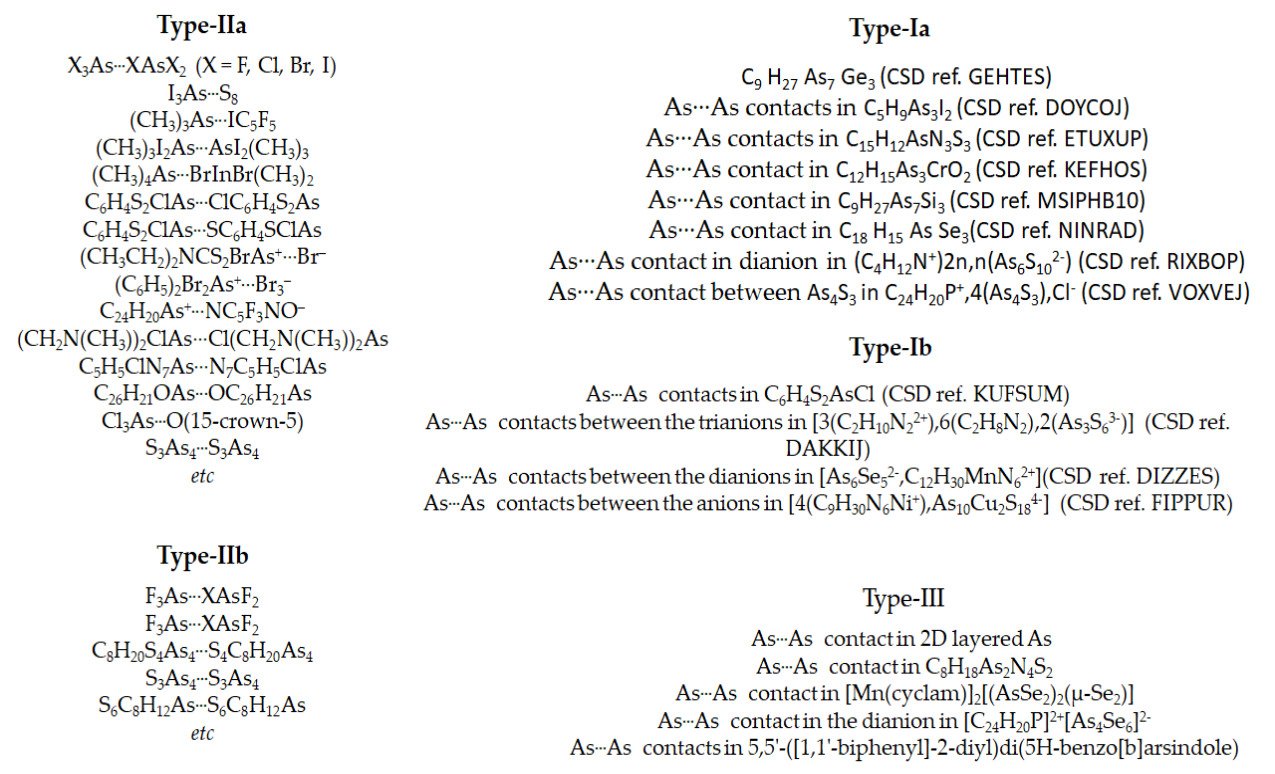
Examples of chemical systems featuring As-centered Type-I, -II and -III interactions. CSD ref. codes are shown in some selected occasions. See Scheme 1 for angular details involved with each interaction type.

Some typical chemical systems featuring As-centered noncovalent topologies shown in Scheme 1 are exemplified in Scheme 2. Some of these systems have been presented in Section 5 below as illustrative systems to display the typical nature of the bonding modes involved.

## 4. Computational Details

Because we were interested in the identification of specific regions on the surface of monomeric entries that display directional features when they find themselves in close proximity to a neighboring entity in a crystal lattice, we performed an analysis of the electrostatic surface potentials of several of these molecular entities as found in the crystal structures. Our specific goal was to identify the electrophilic and nucleophilic regions on them and then demonstrate the way such regions cooperate attractively with each other when in close proximity, thus providing stability to the crystal lattice. 

We optimized the geometry of the selected isolated molecular entities in the gas phase using the Gaussian 16 program package [90] with DFT at the *ω*B97XD level of theory [91] and/or Møller–Plesset’s second-order perturbation theory (MP2) [92]. 

For reasons described below, and depending on the size of the molecular entity and type of atoms involved, basis sets such as ATZP, CC-pVTZ, Aug-CC-pVTZ/Aug-CC-pVTZ-PP, and def2-TZVPD were chosen, and were obtained from the basis set exchange library [93,94]. The calculation of the MESP was performed using the wavefunctions generated on the fully relaxed geometries of the monomeric entities using the AIMAll [95] and Multiwfn [96] codes. The ECP basis set Aug-CC-pVTZ-PP was centered on atom I.

The electrostatic potential *V(r)* that the electrons and nuclei of a molecule create at each point *r* in the surrounding space is given by Eqn 1, where *Z_A_* is the charge on nucleus *A* located at *R_A_*, *ρ(r)* is the charge density function of the molecular entity, and *V(r)* is a physical property that was determined computationally at the same level of theory mentioned above.

(1) $$V\left(r\right)=\sum_{A}\frac{Z_{A}}{\left|R_{A}-r\right|}-\int^{\;}\frac{\rho \left(r^{\prime }\right)dr^{\prime }}{\left|r^{\prime }-r\right|}$$

Since the choice of an isoelectron density envelope on which to compute the electrostatic potential is arbitrary, one can characterize a surface molecular property at any contour such as 0.001 or 0.002 a.u. (electrons/bohr^3^) of the total electronic density function *ρ*(*r*). The choice of a 0.001/0.002 a.u. envelope has been recommended by Bader et al. [97,98] and others [99] since it encompasses at least 95% of the electronic charge and could yield physically reasonable molecular “dimensions”; it remains, nevertheless, an arbitrary choice. 

The sign and magnitude of the local most minimum and maximum of potential, represented by *V_S,min_* and *V_S,max_*, respectively, computed on the isoelectron density surfaces of the monomer entities were used to characterize specific regions on the surfaces of these molecular entities that are either electrophilic or nucleophilic [59,60,100,101]. An electrophilic region was identified when *V_S,min_* > 0 or *V_S,max_* > 0. Similarly, the nucleophilic regions [38,102,103] were characterized when *V_S,min_* < 0 or *V_S,max_* < 0. An attractive interaction between two atomic basins (intermolecular or intramolecular) was recognized when a region of an atom or fragment in a molecular entity with a positive *V_S,min_* (or *V_S,max_*) is in close proximity to that with a negative *V_S,min_* (or *V_S,max_*). A σ-hole interaction was recognized when a positive *V_S,max_* on atom A along the R–A bond extension was observed in close proximity to a negative site described by a negative *V_S,min_* or *V_S,max_*. Similarly, a π-hole interaction was recognized when a positive *V_S,max_* (or *V_S,min_*) on an atom A, or on a fragment, in a molecular entity was observed in the close vicinity of a negative site described by a negative *V_S,min_* or *V_S,max_*. 

Because we were interested in the identification and subsequent characterization of arsenic-centered pnictogen bonding within or between interacting monomer entities, we have used monomer entities in some selected crystals to analyze the pro-molecular charge density based isosurfaces, calculated within the pro-molecular framework of IGM-*δg* [80,81]. We did so as to confirm chemical bonding interactions revealed using MESP and inter- and intramolecular geometries since this method has proven to be useful in providing an insightful local description into the source of the atomic or fragmental domains causing the interaction [38,102,103,104,105]. Within the framework of IGM, pro-molecular atomic electron densities are summed up and the associated atomic gradients do not interfere. This is achieved by using absolute values for summing the atomic gradients and rejecting any electron gradient contragradience feature. The resulting total gradient |∇*ρ*^IGM^| is then an upper limit of the true gradient, and the difference between them, *δg*, quantifies the net electron density gradient collapse due to interactions. This means that the *δg* descriptor identifies the presence of opposite signs in the components of the total electron density gradient |∇*ρ*(r)| due to interactions. IGM automatically separates intra- and inter-fragment interactions in a molecular entity, which can then be plotted in 2D or in 3D (isosurface volumes) to reveal the presence of inter- or intramolecular interactions. The shape of the isosurface volumes can be utilized to infer localized or delocalized interactions between interacting domains; the colors of these volumes in blue and green represent strong and weak attractions, respectively, while red represents a repulsive interaction. 

Analysis and drawing of geometries of various molecular entities and crystals were performed using the Mercury 4.0 [106], Gaussview 5.0 [107], and VMD [108] suite of programs. 

## 5. Arsenic in Crystals

Arsenic poisoning, due to environmental, occupational, accidental, or deliberate exposure, is a significant health risk [109,110,111,112,113,114]. However, its role in thin-film photovoltaic design has been demonstrated in a significant number of studies. For instance, arsenic telluride (As_2_Te_3_) [115] has been extensively investigated as a semiconductor; the *α*-phase of the system (space group *C2/m*) possesses sufficient optical absorption in the 1500 to 3700 nm region [116] and a band gap transition energy of 0.46 eV [117]. Arsenic triselenide, As_2_Se_3_, is another interesting system for use in dielectric metasurfaces because it has a high linear refractive index, *n* = 2.8 and an exceptionally high optical nonlinearity (*n^2^* < 900 × that of silica). Its band gap is 1.8 eV and it possesses a wide transmission window, spanning from approximately 1500–14000 nm, making it useful for applications in the shortwave infrared into the longwave infrared [118]. An alloy of gallium arsenide and gallium phosphide, GaAs_1−*x*_P*_x_*, has been used for manufacturing red, orange, and yellow light-emitting diodes [119]. Gallium arsenide (GaAs) is one of the most widely investigated Group 13–15 direct semiconducting systems with many technological applications including in transistors [120] solar cells and detectors [121], light-emitting devices [122], spintronics [123], and etching [124]. Many studies—both experimental and theoretical—have investigated highly efficient arsenic-doped alloys for technological applications. For example, arsenic-doped cadmium telluride thin films show enhanced hole density and lower dopant diffusivity leading to 20.8% efficient solar cells [125].

Our search of the motif “R–As” in the CSD, which catalogues organic and organometallic crystals, shows that 7064 crystals have been deposited that contain arsenic. As is either (formally) positive, neutral, or negative in these crystals.

The geometric data obtained from the CSD searches were statistically analyzed to infer the range of intermolecular distances and directional features for a variety of donor sites for the arsenic bond. The search was limited to intermolecular distances between 2.6 and 4.2 Å, and bond angles between 130° and 180°. Only single crystals were selected that were free of errors and distortions, and that had an *R*-factor ≤ 0.1. The geometric fragment R–As···D–R’/ R–As···D was chosen (R’ = any element; R, D = elements of Groups 15, 16, and 17 and include carbon in aromatic rings; the bond between D and R’ was of any type). The upper limit of the intermolecular distance was chosen depending on the sum of the vdW radii of As and D.

What follows is a selected set of examples containing As, where As is (formally) positive, neutral, or negative, featuring a variety of As-centered noncovalent and covalent interactions in which As acts as either as a donor or an acceptor of charge density. When the surface of As carries a positive site (an acceptor of charge density) and is involved in an engagement with a negative site, we recognize such an attraction between the bonded atomic basins as an arsenic bond.

### 5.1. Arsenic Trihalides and Related Structures

Our focus is on simple As-based chemical systems to explore fundamentally important situations where covalently bound arsenic attracts negative sites in interacting partner molecules, leading to—or at least assisting in—the formation of crystalline materials. As a starting point, we have chosen here the arsenic trihalides, AsX_3_ (X = F, Cl, Br, I).

**Figure 1:**
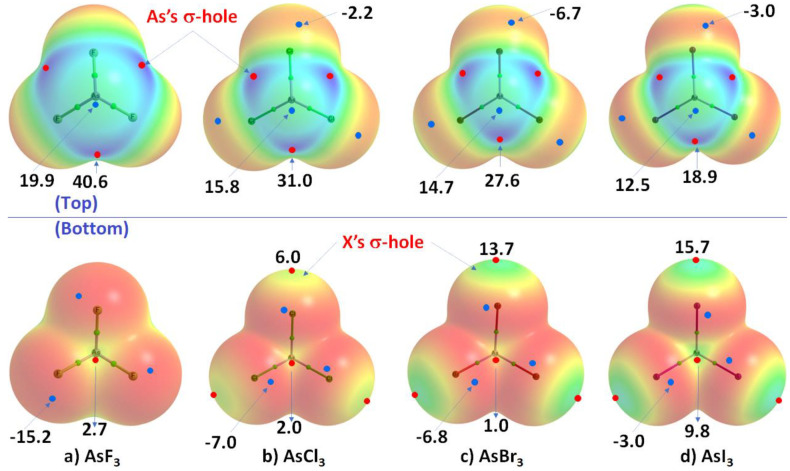
The ωB97XD/ATZP calculated 0.001 a.u. isodensity envelope mapped potential on the electrostatic surfaces of AsX_3_ (X = F, Cl, Br, I) molecules: (**a**) AsF_3_; (**b**) AsCl_3_; (**c**) AsBr_3_; and (**d**) AsI_3_. Selected local most maximum and minimum of potential values represented by *V_S,max_* and *V_S,min_* (marked by tiny circles in red and blue, respectively) are shown in kcal mol^−1^. Two views of each MESP graph for each molecule are displayed: (top) coordinated As faces the reader; (bottom) the three X atoms forming a triangular structure faces the reader.

Before exploring the bonding modes of AsX_3_, we examined their molecular electrostatic surface potentials to provide insight on specific regions of the molecular entities that assist in the formation of crystals. The ωB97XD/ATZP level MESP models of AsX_3_ are shown in Figure 1. The QTAIM-based [126] molecular graphs obtained at the same level of theory are superimposed to clarify the connectivity between the atomic domains responsible for the skeletal framework of AsX_3_ as well as some other systems considered for MESP analysis.

The electrostatic surface of As in AsX_3_ is positive along the outer extensions of the X–As bonds, regardless of the identity of X (Figure 1). This is a *σ*-hole on As; there are three such equipotential *σ*-holes. Unsurprisingly, the value of *V_S,max_* associated with the *σ*-hole on As is largest (40.6 kcal mol^−1^) in AsF_3_ and decreases as X changes from F through to I, reaching the smallest value of 18.9 kcal mol^−1^ in AsI_3_. This is consistent with the decreasing electronegativity and electron-withdrawing capability down the halogen series. This feature suggests that the reactivity of As in AsX_3_, especially in coulombic engagements, should increase as the electronegativity of X increases when in close proximity with the negative sites of a neighboring molecular entity.

In terms of a simple Lewis structure of AsX_3_, one should expect a lone pair on the surface of As along the outer extension of the *C_3v_* axis, accounting for the trigonal pyramidal structure of the molecules. Formally, the electronic configuration of As^3+^, [Ar] 3d^10^ 4s^2^, would have the lone pair in a symmetric *s* orbital. The presence of a stereochemically active lone pair in As^3+^ is well-known (for example [127,128]). However, the MESP model does not show the presence of a lone pair on As in AsX_3_, although it does predict a local most minimum of potential on As along the outer extension of the *C_3v_* axis. As shown in Figure 1 (see Top panel), the *V_S,min_* associated with it decreases in the series from AsF_3_ (19.9 kcal mol^−1^) through to AsI_3_ (12.5 kcal mol^−1^).

F is significantly less polarizable and more electronegative than the other halogens. On the empirical scale of Choudhary, Ranjan & Chakraborty (CR & C) [129], the polarizability *α*(CR & C) in the halogen series follows the order: F = 0.46, Cl = 1.02, Br = 1.67, and I = 1.97. Clearly, the bonding of F with As in AsF_3_ does not allow it to conceive a *σ*-hole on its surface along the As–F bond extension, as observed in NF_3_ and PF_3_ along the N–F [68] and P–F [67] bond extensions, respectively. The (marginally) less electronegativity (*χ*(Pauling) of As = 2.18, P = 2.19, N = 3.44; *χ*(Allen) As = 2.211, P = 2.253, N = 3.610) and more polarizability of As (*α*(CR & C) = 1.97, P = 1.34, N = 0.52) has little ability to pull the electron density from F towards the bonding region; this may explain why the *σ*-hole on F along the As–F bond extensions in AsF_3_ is neutral. This is not the case for AsCl_3_, AsBr_3_, and AsI_3_, where there is a positive potential associated with the *σ*-hole on X along the As–X bond extensions. The strength of the *σ*-hole increases as the electronegativity of X decreases and its polarizability increases (Figure 1, Bottom), and the *σ*-hole is surrounded by negative potentials (Figure 1, Top).

An inconsistency in the potential associated with the central region of the triangular face formed by the three halogen atoms in each AsX_3_ molecule is notable as there is no trend in the *V_S,max_* values across the series (AsI_3_ > AsF_3_ > AsCl_3_ > AsBr_3_). To verify whether this was an artefact of the basis set used, we recalculated the *V_S,max_* and *V_S,min_* values using the def2-TZVPPD basis set, in conjunction with MP2(full) and ωB97XD. The results are summarized in Table 1.

Both methods produced very similar trends and the values of the *σ*-holes on As and X predicted using the ATZP basis set were retained. However, *V_S,max_* values associated with the central region of the triangular face formed by the three halogen atoms in AsF_3_ molecule was found to be the largest and the trend in the *V_S,max_* values across the series now follows the order AsF_3_ > AsI_3_ > AsBr_3_ > AsCl_3_, revealing a dependency of the magnitude of potential on the size and quality of the basis set used.

### 5.2. The As···X (X = Halogen) Arsenic Bonds

#### 5.2.1. The Crystal Structure of AsF_3_

**Figure 2:**
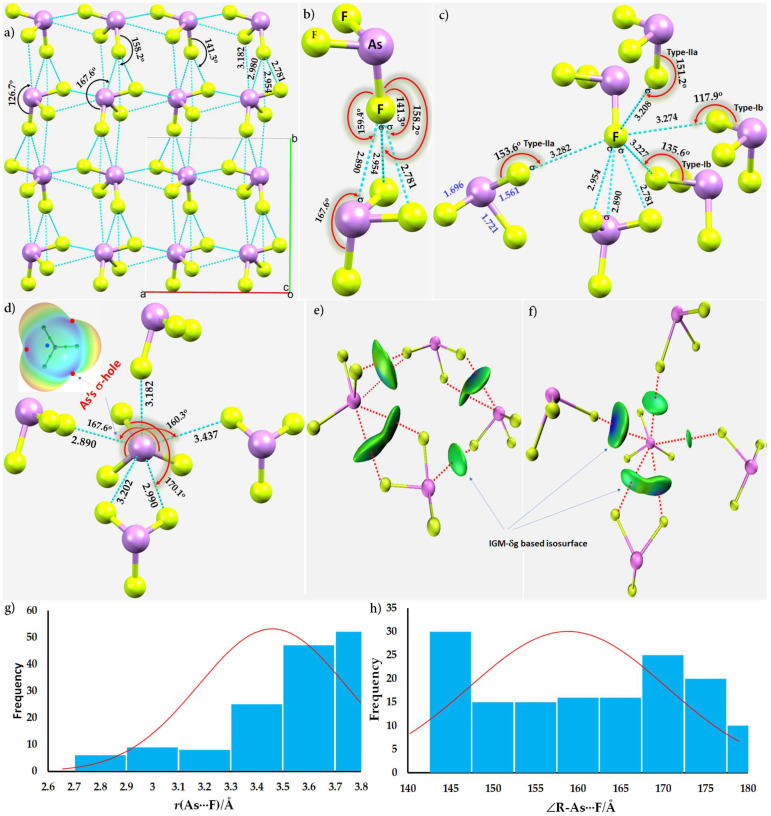
(**a**) The nature of intermolecular bonding topologies between the interacting molecules in the 2 × 2 × 2 supercell structure of the AsF_3_ crystal (ICSD ref. code 35132). (**b**) The specific *σ*-hole interactions between a pair of adjacent AsF_3_ molecules in the crystal. (**c**) Nature of hepta-furcated topology of covalently bound F in AsF_3_ with the neighboring molecules. (**d**) Nature of penta-furcated topology of covalently bound As in AsF_3_ with the neighboring molecules, with the insert representing the MESP of AsF_3_ (see Figure 1a). (**e**) IGM-δ*g* level isosurface topologies between the four AsF_3_ molecules as in the unit-cell of the crystal. (**f**) IGM-δ*g* level isosurface topologies between the AsF_3_ molecules, showing the occurrence of As···F arsenic bonds in the crystal. (**g**,**h**) Histograms showing the distribution of As···F close contacts in 62 crystals from a CSD search. The As···F intermolecular distance *r* (and intermolecular angle ∠R–As···F) in the range of 2.6–3.8 Å (140–180°) was used as a geometric criterion during the CSD search, where R represents any atom. Bond lengths and angles are shown in Å and degrees, respectively. Regions characteristic of *V_S,max_* and *V_S,min_* in the insert in (**d**) are marked by tiny circles in red and blue, respectively. The red curve in (**g**,**h**) represents the normal distribution.

The crystal structure of arsenic trifluoride, AsF_3_, reported in 1982, is shown in Figure 2a [130]. Our analysis indicates that each F in a given AsF_3_ unit is involved in seven intermolecular contacts with AsF_3_ units, and this can be seen in Figure 2a,b. Three of them have bond distances in the range of 2.9–3.0 Å. The remaining four are in the range of 3.20–3.30 Å. The first three are less than the sum of the van der Waals radii of two fluorine atoms, 2.92 Å. In this case, the arsenic-bound F atom probably serves as a donor for two *σ*-hole bonds (Figure 2b), and is engaged in two *σ*-hole interactions with two F sites in a neighboring AsF_3_ unit, leading to the formation of two, non-equivalent Type-IIa and Type-IIb F···F halogen···halogen bonds (∠As–F···F = 158.2° and 141.3°, respectively). At the same time, it acts as an acceptor of a *σ*-hole-centered Type-IIa arsenic bond from the As on the same neighboring AsF_3_ unit with an intermolecular bond distance of 2.890 Å, much shorter than the sum of the van der Waals radii of As and F (3.34 Å). The angle of the latter interaction is quasi-linear, with ∠F–As···F = 167.6°. The first two interactions are a forced consequence of the latter, which is undoubtedly coulombic in origin. One might conclude that the first two interactions of F are a result of σ-hole interactions since they appear along the extension of the As–F σ-bonds, even though the MESP model fails to detect a σ-hole on the surface of the F atom in AsF_3_. The development of a *σ*-hole on F could be induced by the electric field of the surrounding F atoms of the partner molecule that is in close proximity, as advanced in some previously reported studies [37,131]; or it could be the forced consequence of the As···F primary interactions.

In addition to the three interactions described above, we also observed that the same F site in AsF_3_ acts as an acceptor of another two fluorine-centered *σ*-hole bonds, Figure 2c. These are longer, with *r*(F···F) = 3.208 and 3.282 Å. They result from attractions between the *σ*-hole regions on the As–F bonds of two surrounding AsF_3_ molecules and the fluorine marked F in the third molecule. They are directional (Type-IIa), with ∠As–F···F = 151.2° and 153.6°, respectively. The remaining two As–F···F interactions follow a Type-Ib topology of bonding, with intermolecular bond distances of 3.227 and 3.274 Å (Figure 2c). They are significantly nonlinear, with ∠As–F···F = 135.6° and 117.9°, respectively.

The As site in AsF_3_ along the three F–As bond extensions serves as a center for donating five arsenic bonds, as seen in Figure 2d. Three develop along the three F–As bond extensions and are quasi-linear with bond distances of 2.890, 2.990, and 3.437 Å; the other two, with bond distances of 3.182 and 3.202 Å, appear off the F–As bond axes (F–As···F = 137.3° and 131.8°, respectively). They are nonlinear arsenic-centered pnictogen bonds, and follow a Type-IIb topology of bonding. While the Type-I/Type-II halogen···halogen bonding and Type-I/Type-II As···F pnictogen-bonding interactions are clearly present, the latter seem to be stronger than the former; the fomer appear to have developed as a consequence of the primary Type-IIa As···F interactions.

Our IGM-δ*g* level analysis, shown in Figure 2e,f, suggests that the detailed network of Type-I/Type-II halogen···halogen bonding and Type-I/Type-II As···F pnictogen-bonding interactions shown in Figure 2c,d cannot be fully appreciated by considering only the molecular domains within a unit-cell of the AsF_3_ crystal. Figure 2e provides insight into the As···F and F···F interactions between the AsF_3_ molecules in the unit-cell of the AsF_3_ crystal. The first are indicated by the green and bluish-green isosurface volumes between the As and F atomic basins that signify attractive interactions between bonded atomic basins. Similarly, Figure 2f confirms the five-fold intermolecular bonding interactions formed by each As site in the AsF_3_ crystal.

The local bonding features in the AsF_3_ crystal shows that the pnictogen atom has the ability to form multiple pnictogen bonds (five, in this case) with neighboring molecules; thus, As in AsF_3_ is nine-fold coordinate. This finding appears to contradict the contention of others that the number of pnictogen bonds between a Group 15 element and a set of bases is dependent on the strength of the base [34]. They showed that a weak base like NCH might be limited to only two pnictogen bonds with AsF_3_, whereas a stronger base like NH_3_ can engage in three and even four bonds in certain circumstances. Our analysis of the crystal structure of AsF_3_ does not support the generalization. In the following two examples, we show that the conclusion we arrived at is also true in the case of both AsCl_3_ and AsBr_3_; this is indeed a result of packing forces acting on the system in the crystalline phase.

The sum of the vdW radii of As and F is 3.34 Å, (*r_vdW_* (As) = 1.88 Å and *r_vdW_* (F) = 1.47 Å). Our initial search of the CSD for R–As···F that restricted the As···F intermolecular bond distance and ∠R–As···F intermolecular bond angle to ranges of 2.6–3.4 Å and 140–180° found only 19 hits. We then expanded the bond distance range to 2.6–3.8 Å without changing the angular constraint. Our search for the R–As···F–R’ (and R–As···F) close contacts found 64 (66) hits, with the latter comprising a total of 160 close contacts. We found four crystals with 11 false contacts. For example, the purported As···F close contact in [C_6_H_5_AsF_19_P_2_Pt][AsF_6_] (CSD ref. ZIBKEC) [132] is between As in AsF_6_^–^ and F in the cation. Hence, each and every supposed contact has to be critically examined to eliminate such outliers. This was so in the cases of As···Cl, As···Br, As···I, and As···S contacts as well (*vide infra*).

In the majority of the 62 crystals, the lateral portion of F in a given building block was found interacting with the axial site of covalently bound As, although, in some cases, the axial portion of covalently bonded F was also in engagement with the As site in a partner entity. We did not investigate the details of whether this is the result of fluorine-centered halogen bonding, F···As. Along with the long As···F contacts, multiple primary and secondary interactions are present, causing the interaction to become nonlinear.

The normal distribution of As···F bond distance *r* and bond angle ∠R–As···F for a total of 147 As···F close contacts from 62 crystals is shown in Figure 2g,h. As can be seen, the peaks of the normal distributions are centered at 3.45 Å and 159°, respectively. The maximum occurrence of As···F close contacts were found in the range of 3.3–3.7 Å while the angles were nearly equally distributed between 145 and 170°. Clearly, the upper limit of the bond distance range suggests that the “less than the sum of the van der Waals radii” cannot be taken as a mandatory criterion to search for noncovalent As···F interactions.

#### 5.2.2. The Crystal Structure of AsCl_3_

Glay et al. [130] synthesized and examined the crystal structure of arsenic trichloride, AsCl_3_, and later [133] used X-ray diffraction (176–250 K) to re-examine the crystalline state, as well as wide-angle X-ray scattering (WAXS) to examine the liquid state of the compound. The molecular structure of AsCl_3_ is very similar in both the solid and the liquid phases, and indeed in the gas phase [134].

**Figure 3:**
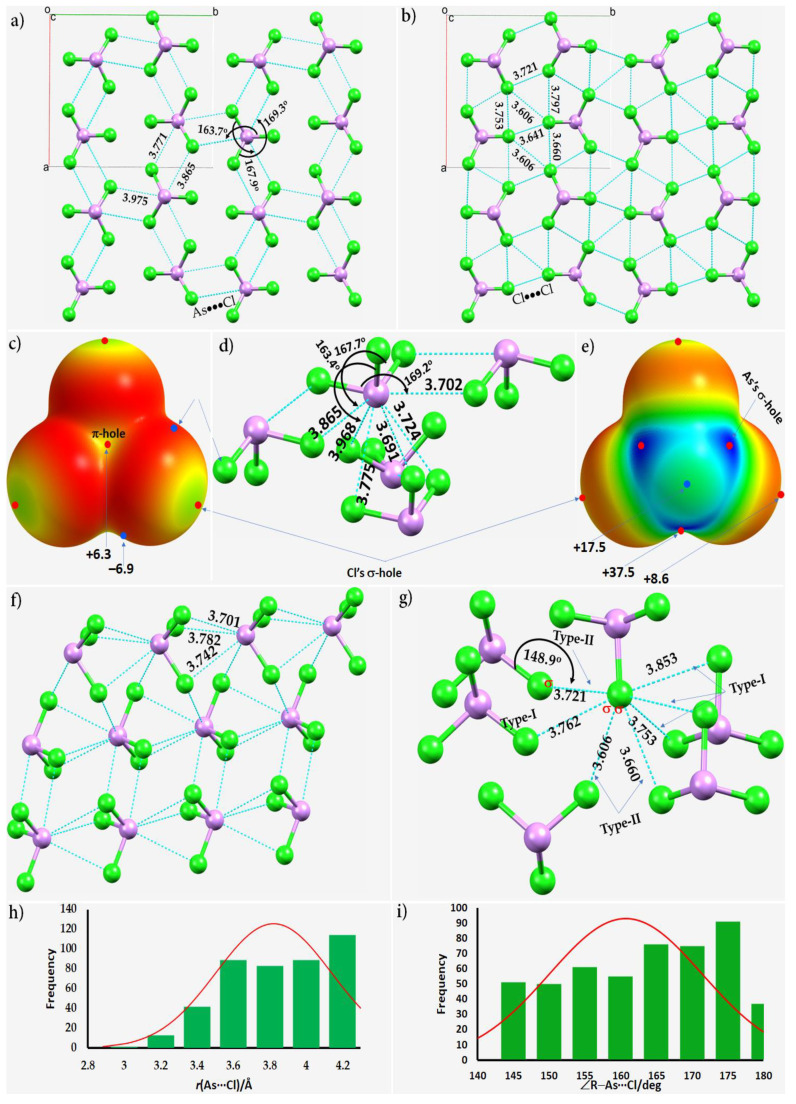
(**a**,**b**) Illustration of the geometrical aspects of the Cl–As···Cl and As–Cl···Cl pnictogen and halogen···halogen bonding interactions in crystalline AsCl_3_ l (ICSD ref. 280796). (**c**) ωB97XD/aug-cc-pVTZ computed 0.0015 a.u. isoelectron density mapped electrostatic potential on the surface of arsenic trichloride (AsCl_3_), showing the positive and negative regions on the covalently bonded Cl sites and the location of a π-hole along the extension of the *C_3_v* axis. (**d**) Illustration of the hexa-furcated interaction topology of covalently bound arsenic in crystalline AsCl_3_ (ICSD ref. 35133). (**e**) ωB97XD/aug-cc-pVTZ computed 0.0015 a.u. isoelectron density mapped potential computed on the 0.0015 a.u. isodensity envelope of AsCl_3_, displaying the nature of the potential along the outer extensions of the As–Cl and Cl–As bonds, and the π-hole on As along the extension of the *C_3_v* axis. (**f**) Illustration of (As)π ···π(As) interactions, caused by nonlinear As···Cl interactions (ICSD ref. 280796). (**g**) Illustration of the penta-furcated interaction topology of covalently bound Cl in crystalline AsCl_3_ (ICSD ref. 280796). (**h**,**i**) Histograms showing the distribution of As···Cl close contacts in various crystals resulted from a CSD search. The As···Cl intermolecular distance (and intermolecular angle ∠R–As···Cl) in the range of 2.6–4.3 Å (140–180°) was used as a geometric criterion during the CSD search, where R represents any atom of the periodic table. *V_S,max_* and *V_S,min_* values marked by tiny circles in red and blue, respectively, in (**c**,**e**) are shown in kcal mol^−1^. Bond lengths and angles are shown in Å and degrees, respectively. The red curve in (**h**) and (**i**) represents the normal distribution.

AsCl_3_ crystallizes in the *P*2_1_2_1_2_1_ orthorhombic space group. The 2 × 2 supercell structure is shown in Figure 3a,b. Our analysis of the geometry of the crystal indicates that there are a number of intermolecular contacts between AsCl_3_ molecules along the Cl–As and As–Cl bond extensions, as shown in Figure 3a,b, respectively. In particular, the three (Cl–)As···Cl intermolecular bond distances (bond angles) that originate along the three Cl–As bond extensions of an AsCl_3_ molecule are 3.865 Å (167.9°), 3.975 Å (163.7°), and 3.771 Å (169.3°), in the crystal reported in 2002 (ICSD ref. 280796) [133]. These differ slightly from the earlier report (ICSD ref. 35133) [130], where the equivalent values are 3.702 Å (169.2°), 3.968 Å (163.4°), and 3.865 Å (167.7°). The development of these intermolecular interactions between the AsCl_3_ units in the crystal is expected when the negative lateral sides on the Cl atom around the As–Cl bond extension in a given AsCl_3_ unit is in close proximity to the positive *σ*-holes (*V_S,max_* = +37.5 kcal mol^−1^) localized on As along the Cl–As bond extensions in a neighboring AlCl_3_ unit (cf. Figure 3c–e).

Because the electrostatic surface of As in AsCl_3_ is entirely positive (Figure 3e), it is also involved attractively with the negative portions around the Cl atoms on neighboring molecules. This results in the formation of three additional As···Cl and As(π)···As(π) pnictogen-bonded interactions (Figure 3f). The As···Cl interactions are all nonlinear (∠Cl–As···Cl between 125° and 135°) and are partly arising from As(π)···As(π) pnictogen interactions. Although the three As···Cl intermolecular interactions feature a Type-Ib topology of bonding, they are Type-IIb pnictogen bonds since they are the result of the attraction between sites of opposite electrostatic potential on the interacting atoms. Hence, As in AsCl_3_ acts as a hexa-furcated center to donate pnictogen bonds.

The covalently bound Cl site in AsCl_3_ features six or seven interactions within the distance range of 3.5–4.0 Å. The latter are shown in Figure 3g. There are three *σ*-hole-centered As–Cl···Cl halogen-bonded interactions, indicated by an ∠As–Cl···Cl angle and the symbol ‘*σ*’ on Cl. The intermolecular distances corresponding to these three interactions are either slightly longer or slightly shorter than the vdW radii of two Cl atoms, 3.64 Å. The remaining four Cl···Cl intermolecular contacts are not σ-hole-bonded interactions. They are pronouncedly nonlinear and follow a Type-Ib topology of bonding; the attraction between the negative sites on the Cl atoms in the interacting molecules cause their development. We expect that the strength of most of the Type-Ib Cl···Cl intermolecular interactions fall in the weak to vdW range.

The complicated intermolecular bonding topologies between the interacting molecules in crystalline AsCl_3_ locally form an irregular triangular arrangement, an X_3_ bonding topology, between the Cl atoms of three neighboring AlCl_3_ molecules (Figure 3b) with angles of 60.7°, 60.1°, and 59.2°. When four of them interact, the resulting Cl_4_ bonding arrangement has a distorted rhombohedral shape(Figure 3b).

We note that the X_3_ bonding topology is very similar to that found in crystalline PI_4_ [67] and in crystal materials containing halogens [135]; these interactions have been referred to as synergic interactions and are recognized to be the result of cooperative interactions between halogen–halogen-bonded X_3_ or X_4_ (X = F, Cl, Br, I) synthons [136,137,138,139,140,141]. This hypothesis needs to be tested in a future study on simplified model clusters of AsCl_3_, *inter alia*, as has been done elsewhere [135]. In that study, the authors examined 44 crystals deposited into the CSD featuring the X_3_ synthon (X = Cl, Br, I) to verify whether the Type-IIa halogen–halogen contacts forming the synthon display any cooperativity. Of the 44, only two structures containing the I_3_ synthon showed a very weak or weak synergy, i.e., having a cooperative effect stronger than −0.40 kcal mol^–1^. The crystal structure of CHI_3_ was found to have the most pronounced cooperativity of all the systems investigated, amounting to about 10% of the total interaction energy.

We initially used the intermolecular bond distance and intermolecular bond angle ranges of 2.6–3.8 Å and 140–180° as geometric criteria to search for As···Cl close contacts in crystals deposited into the CSD. These criteria led to 112 single crystals that feature 228 As···Cl close contacts. In the great majority of them, covalently bonded As is attractively engaged with the lateral portions of Cl in a neighboring molecule. While inspecting all these results, we found all As···Cl contacts to be a genuine contact, yet many are missing because of the constraint imposed on the bond distance range. For example, the crystal ([C_16_AlCl_12_F_24_O_4_][C_14_H_10_AsBiMo_2_O_4_](CH_2_Cl_2_)_0.5_ (CSD ref: CALFAA [142]) contains an As···Cl interaction at a distance of 4.136 Å and an Mo–As···Cl bond angle of 179.9°. We then extended the bond distance range to 2.6–4.3 Å with the same angular range as the constraint of our search; this produced 244 results with 496 close contacts. An inspection of these showed 11 crystals containing 18 false contacts (*vide supra* for an explanation); these were discarded. Many of the remaining 478 As···Cl close contacts appear to be a consequence of H···Cl hydrogen bonds. The histograms shown in Figure 3h,i include all contacts. The peaks of the normal distribution occur around 160° and 3.8 Å for the bond angle and bond distance, respectively. Most As···Cl close contacts were found in the range of 3.5–4.2 Å, skewed towards the higher end of the range, while the ∠R–As···Cl angles were fairly evenly distributed between 145° and 175°. Again, it is event that many close contacts lie outside the limits of the “sum of the van der Waals radii” criterion and appear to be a consequence of other primary/secondary noncovalent interactions in these crystal structures. Once again, a strict adherence to the “less than the sum of the vdW radii” concept would have missed these contacts.

#### 5.2.3. The Crystal Structure of AsBr_3_

**Figure 4:**
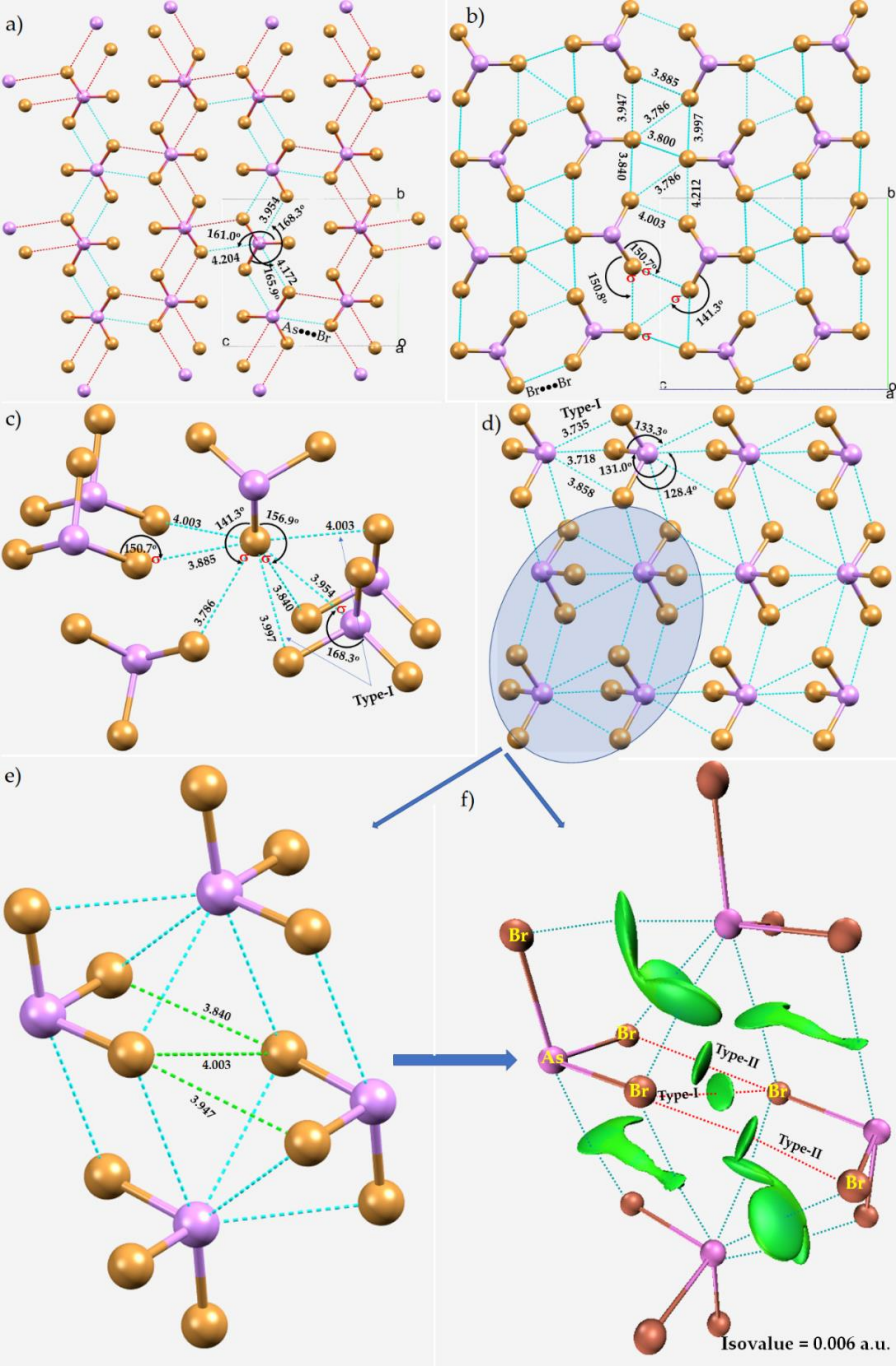
(**a**) The nature of As···Br arsenic bonds in the 2 × 2 × 2 supercell structure of the crystal of arsenic tribromide (AsBr_3_) (ICSD ref. code 26774). (**b**) The nature of Br···Br bonding interactions, showing the emergence of the Br_3_ and Br_4_ synthons in the crystal. (**c**) The local nature of intermolecular bonding modes of Br in AsBr_3_. (**d**) Snapshot of combined interactions along crystallographic *a*-direction, showing lapping parallel As(π)···π(As) and Br···π(As) interactions. (**e**) The local nature of bonding between the four AsBr_3_ units, extracted from (**d**). (**f**)The IGM-δ*g* based isosurface topologies between bonded atomic basins in the cluster of four AsBr_3_ unitsSelected bond distances and angles are in Å and degrees, respectively.

Singh and Swaminathan appear to have been the first to report the crystal structure of arsenic tribromide in 1964 [143]. The same authors reported a refined structure in 1967 [144], although Trotter had reported the structure of the system in 1965 [145]. In all cases, the crystals of AsBr_3_ were orthorhombic, with space group *P*2_1_2_1_2_1_. Trotter’s structure (ICSD ref. 26774) is shown in Figure 4a,b. In the structure, As–Br = 2.36 Å and ∠Br–As–Br = 97.7°. The shortest intermolecular distances between the molecular units were reported to be As···Br = 3.72 Å, Br···Br = 3.79 Å, and As···As = 4.33 Å. Whether or not these interactions represent to pnictogen bonding (or halogen bonding) was not clarified since these terms were not coined during that time.

Our analysis suggests that the modes of intermolecular interactions between the molecules in crystalline AsBr_3_ are very similar to those discussed above for AsCl_3_. There are many Br–As···Br and As–Br···Br intermolecular interactions present. Although the intermolecular bonding modes of As in the crystal are not very complicated, the attractive engagement of the strongly positive *σ*-holes and weakly negative lateral sites on Br along and around the As–Br bond extensions with the negative sites around the lateral sites of Br in the surrounding molecules build complicated topologies of noncovalent interactions. Each Br in a given AsBr_3_ molecule serves as an hepta-furcated Lewis acid/base center for the surrounding partner molecules (Figure 4c). It is involved in attractive engagements with the seven nearest neighboring Br/As sites on five or six neighboring molecules at a time, forming at least six Br···Br contacts and one As···Br contact.

Because of steric crowding of the Br sites in the crystal, the As–Br···Br *σ*-hole-centered interactions observed in this study are all appreciably nonlinear. Each Br accepts two or three σ-hole bonds from the interacting Br sites in neighboring molecules. At the same time, it donates two halogen-centered *σ*-hole bonds. The Br···Br intermolecular distances associated with the first two, shown in Figure 4c, are 3.786 and 3.840 Å, respectively, with angles of interaction of 141.3° and 156.9°, respectively. Similarly, it serves as an acceptor of two *σ*-hole bonds donated by As in neighboring molecules, with bond distances of 3.858 and 3.958 Å (cf. Figure 4b,d). The remaining Br···Br intermolecular distances are typical of Type-I halogen···halogen bonds. The Type-IIa Br···Br contacts locally form triangle-shaped structures (cf. Figure 4b), presumably providing additional strength to the skeletal framework of the crystal. This is similar to that found in the AsCl_3_ crystal (cf. Figure 3b).

Each As site in an AsBr_3_ molecule in the crystal is involved in forming at least three *σ*-hole-centered arsenic bonds with the surrounding Br sites of three neighbors (Figure 4a). The As···Br bonds are not equivalent, with distances (∠Br–As···Br) of 3.954 Å (168.3°), 4.172 Å (165.9°), and 4.204 Å (161.0°), although all are reasonably directional (Figure 4d,e). Other than these three Type-IIa pnictogen-bonding interactions formed by the *σ*-holes on the Br–As extensions, the same site is also involved in forming another three pnictogen bonds with each AsBr_3_ neighbor, in which the negative sites on Br atoms are in an attractive engagement (cf. Figure 4d). They are bent, Type-IIb, with As···Br bond distances (∠Br–As···Br) of 3.718 (133.0°), 3.735 (133.3°), and 3.858 Å (131.0°).

**Figure 5:**
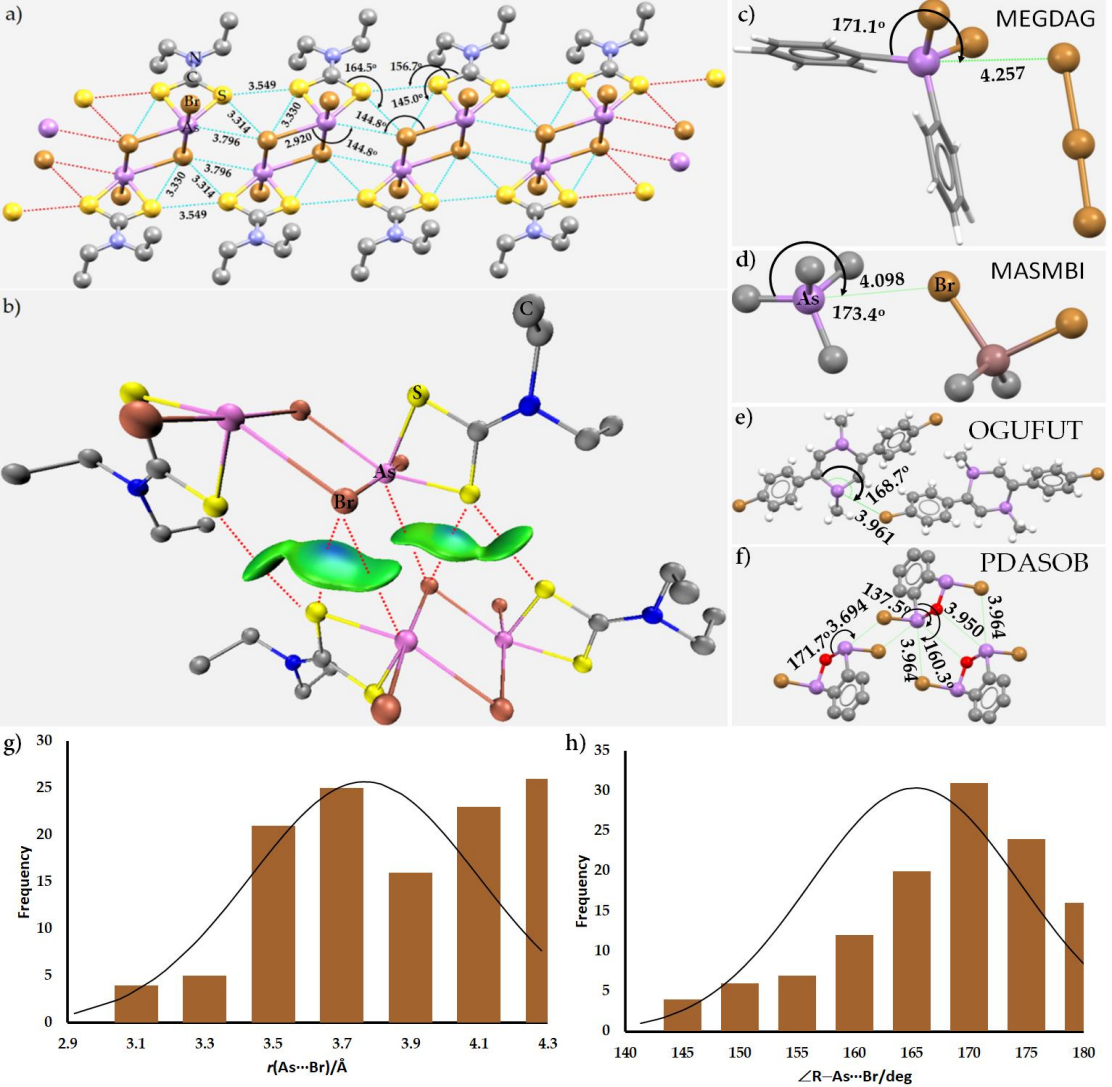
(**a**) The typical nature of intermolecular interactions in the compound [(CH_3_CH_2_)_2_NCS_2_AsBr]Br. (**b**) The IGM-δ*g* isosurfaces between a pair of units of [(CH_3_CH_2_)_2_NCS_2_AsBr]^+^. Shown in (**c**–**f**) are the binary arrangements between the building blocks in the crystals dibromo-diphenyl-arsenic tribromide hemikis(dibromine) [147], tetramethylarsonium dibromo-dimethyl-indium(II) [148], (*S,S*)-2,5-bis(4-bromophenyl)-1,4-dimethyl-1,4-dihydro-1,4-diarsinine [149], and o-phenylenediarsine oxybromide [150], in which the As···Br close contact distances are longer than the vdW radii of As and Br atomic basins, 3.74 Å. (**g**,**h**) Histograms showing the distribution of 120 As···Br close contacts in 52 crystals from a CSD search. The As···Br intermolecular distance *r*(As···Br) (and ∠R–As···Br) in the range of 2.6–4.3 Å (140–180°) was used as a geometric criterion during the CSD search, where R represents any atom. Selected bond distances and bond angles are in Å and degrees, respectively. Atom type is shown in (**a**,**b**,**d**), and H atoms in (**a**) are omitted for clarity and in (**b**) are missing in deposited structure. CSD reference codes are shown for each case. The red curve in (**h**) are the normal distribution.

The arsenic bonds observed in crystalline AsBr_3_ described above are somewhat shorter than those observed in [(CH_3_CH_2_)_2_NCS_2_AsBr]Br, bromo-(*N,N*-diethyldithiocarbamato)arsenic bromide [146]. There are two such As···Br links between a pair of adducts, Figure 5a, with (*r*(As···Br) = 3.796 and 3.996 Å and they are significantly nonlinear (∠Br–As···Br = 144.8°). These, together with a network of S···Br chalcogen bonds, are responsible for the formation of infinite chain-like sheets. That the [(CH_3_CH_2_)_2_NCS_2_AsBr]^+^ units in the crystal are connected with each other through As···Br and Br···Br links is shown by the IGM-δ*g* isosurfaces shown in Figure 5b. In all cases, the intermolecular distance associated with each noncovalent interaction is less than the sum of the vdW radii of the respective atomic basins (*r_vdW_* (S) + *r_vdW_* (Br) = 3.75 Å; *r_vdW_* (Br) + *r_vdW_* (Br) = 3.72 Å).

We used bond distances in the range of 2.6–4.3 Å and ∠R–As···Br in the range of 140–180° to search for As···Br close contacts. This has resulted a total of 61 hits in a CSD search. We found that there were 19 false contacts (nine crystals) out of a total of 141 closed contacts. In most cases the purported contact was between covalent bound Br in one moiety and F in a AsF_6_^–^ anion. The bond distance and bond angle distributions are shown in the histograms in Figure 5g,h, respectively. As can be seen, the peak of the normal distribution associated with 120 contacts centers around 165° and 3.76 Å for the bond angle and bond distance, respectively. The maximum occurrence of As···Br close contacts were found in the ranges of 3.5–4.3 Å and 155–180° for the bond distances and bond angles, respectively. Clearly, many As···Br close contacts do not strictly obey the “less than the sum of the van der Waals radii” criterion.

#### 5.2.4. The Crystal Structure of AsI_3_

Crystalline arsenic triiodide, AsI_3_, was reported in 1965 [151] and again in 1980 [152]. The crystal structure is rhombohedral. The arsenic atoms are significantly displaced from the centers of iodine octahedra; thus, they have three near-neighbor iodine atoms. Clearly, the crystal structure may be considered to be built up from discrete AsI_3_ molecules. Intermolecular As···I distances of 3.50 Å were noted by Trotter [151], but no view was expressed concerning the possibility of arsenic and halogen-bonding in the crystal since none of these terms were in used at the time.

**Figure 6:**
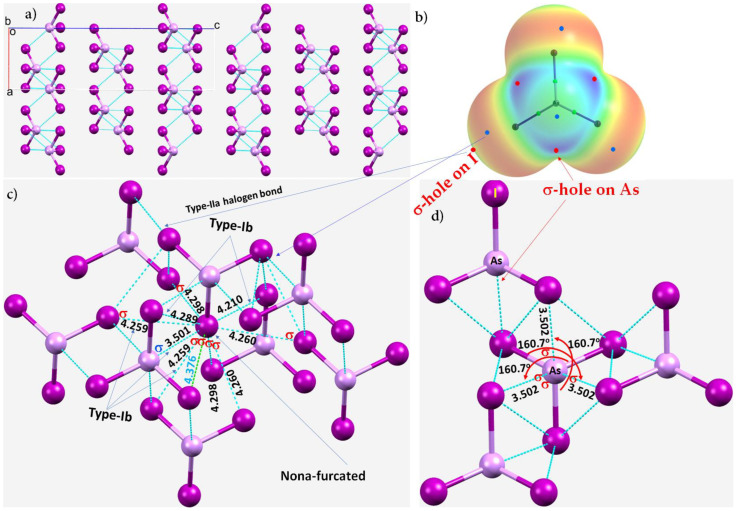
(**a**) The 2 × 2 × 2 supercell structure of the crystal of arsenic triiodide, AsI_3_ (ICSD ref. code 26095), illustrating the likely occurrence of I–As···I intermolecular interactions. (**b**) The MESP model of AsI_3_ as shown in Figure 1d, displaying the way σ-holes on covalently bonded I and As are involved in forming various bonding interactions in the crystal. (**c**) The nature of topology of I···I bonding interactions in the crystal, and the symbol “*σ*” represents the atom donating the *σ*-hole bond. (**d**) The topology of As-centered I–As···I pnictogen-bonding interactions formed by a given molecule of AsI_3_. Selected bond distances and bond angles are in Å and degree, respectively. Surface regions corresponding to *V_S,max_* and *V_S,min_* are marked by tiny circles in red and blue, respectively, in (**b**). Bond lengths and angles are shown in Å and degrees, respectively.

Our analysis of the intermolecular interactions in the crystal is shown in Figure 6. As expected from the results of the MESP model (Figure 1d), each of the three I sites in AsI_3_ acts as a donor of three *σ*-hole bonds, while the negative lateral sites act as donors of electron density. Each I site is an acceptor of four *σ*-hole bonds with intermolecular distances varying between 3.50 and 4.30 Å range (cf. Figure 6b,c). Simultaneously, it is a donor of three or four *σ*-hole bonds. Clearly, covalently bound iodine acts both as an acceptor and a donor of electron density; this arises because the electron density on the electrostatic surface of iodine in AsI_3_ is anisotropic. This analysis leads to the conclusion that each covalently bound I in AsI_3_ is (at least) an octa-furcated center. This is not unexpected since I is more polarizable, has a stronger *σ*-hole, and a larger radius than the other halogens in the AsX_3_ family.

The I···I intermolecular distances associated with the Type-II interactions are greater than the sum of the van der Waals radii of two I atomic basins, 4.08 Å. They are significantly nonlinear, with ∠As–I···I close to either 140° or 152°. Each bonded I site in the molecule is also engaged in forming one or two Type-Ib interactions, with an intermolecular distance in the range of 4.1–4.2 Å (one shown in Figure 6b). Because the bond distances associated with the Type-Ib and Type-IIa interactions are longer than the vdW radii sum, they are probably weak and largely dispersion-driven.

The I–As···I intermolecular interactions in the crystal, where I acts as a pnictogen bond acceptor, are directional (∠I–As···I = 160.7° and *r*(As···I) = 3.502 Å in the AsI_3_ crystal structure, ICSD ref 26095). The corresponding values are 162.3° (159.6°) [162.1°] and 3.462 (3.559) (3.436) Å in the crystal of the same system of that with ICSD refs. of 86495 (56571) (23003). Each As site in AsI_3_ forms three equivalent pnictogen-centered *σ*-hole interactions in each crystal (Figure 6c), although they are not equivalent when all four AsI_3_ crystals are compared.

The average (X–)As···X contact distances in AsX_3_ are 3.170 Å when X = F, 3.870 Å when X = Cl, 4.110 Å when X = Br, and 3.462 Å when X = I, suggesting, based purely on the average contact distances, that these noncovalent interactions are strongest in AsF_3_ and weakest in AsBr_3_. This trend in stability is unusual since the strength of As···X in AsBr_3_ is weaker than that in AsI_3_; this may be revealed in future studies.

**Figure 7:**
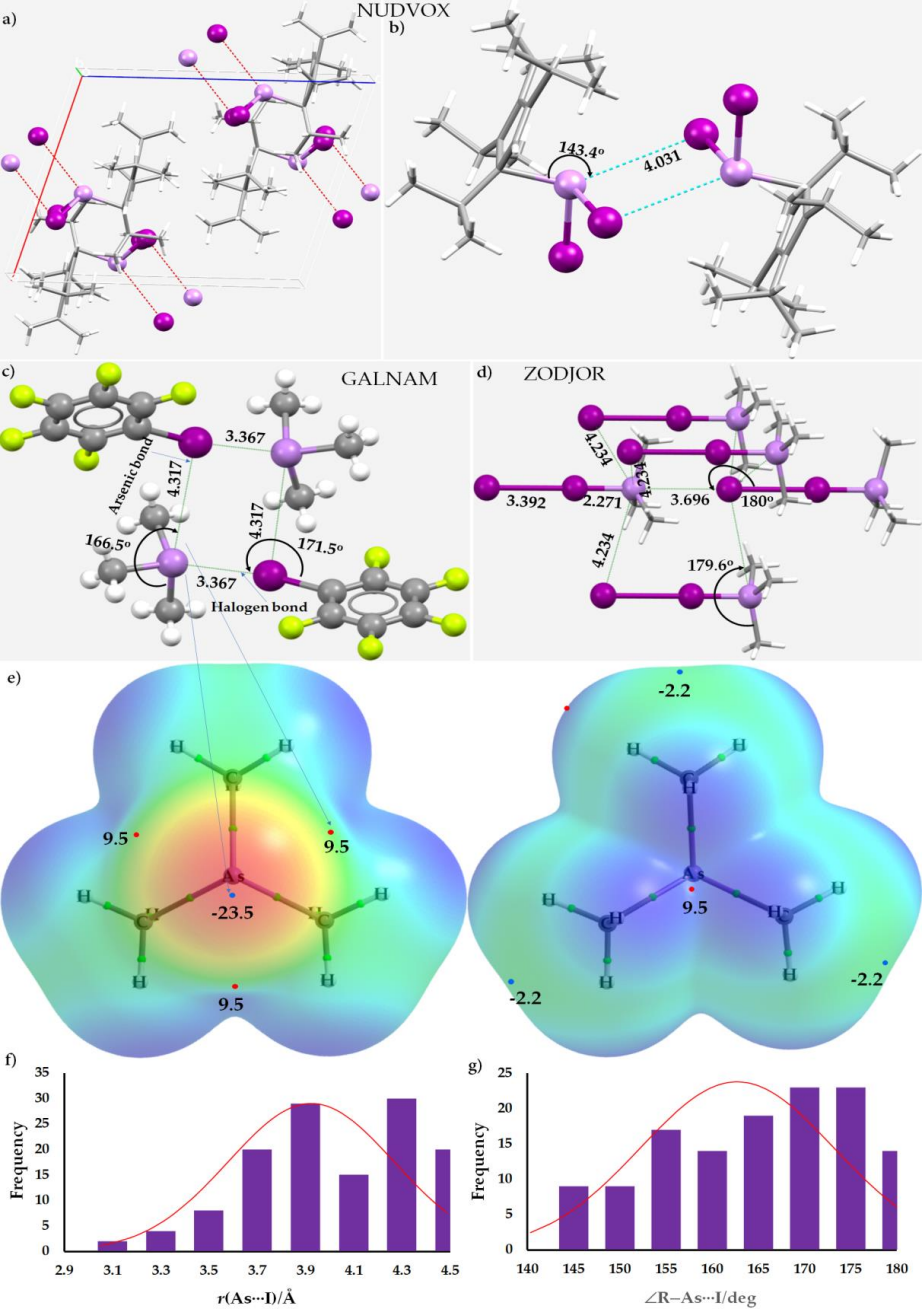
(**a**) The crystal structure of tetraisopropylcyclopentadienyl arsenic(III) diiodide ((TipCpAsI_2_), CSD ref. code NUDVOX). (**b**) Illustration of typical As···I pnictogen bonding (dots in cyan between atoms) in the crystal. Shown in (**c**,**d**) are the arrangement between the building blocks in the crystals of 1,2,3,4,5-pentafluoro-6-iodobenzene trimethylarsane (C_3_H_9_As,C_6_F_5_I) [155] and di-iodo-trimethyl-arsenic (C_3_H_9_AsI_2_) [156] with long As···I pnictogen bonds. (**e**) Two different views of the wB97XD/def2-TZVPPD level 0.001 a.u. mapped electrostatic potential on the surface of the As(CH_3_)_3_ molecule: (Left) As facing the viewer; (Right) opposite side of As facing the viewer. (**f,g**) Histograms showing the distribution of As···I close contacts in various crystals from a CSD search. The As···I intermolecular distance *r*(As···Cl) (and ∠R–As···Cl) in the range of 2.6–4.5 Å (and 140–180°) was used as a geometric criterion during the CSD search, where R represents any atom of the periodic table. Surface regions corresponding to *V_S,max_* and *V_S,min_* values marked by tiny circles in red and blue, respectively, in (**e**) are shown in kcal mol^−1^. Bond lengths and angles are shown in Å and degrees, respectively. Hanging contacts in (**a**) represent pnictogen bonding. The red curve in (**f,g**) represents the normal distribution. CSD codes shown in each case.

The angular characteristic of the As···I arsenic bonds in AsI_3_ is different from that observed in the arsenic dithiocarbamate iodide, As[S_2_CN(CH_2_)_5_]_2_I, where ∠As–I···I = 144.3° and *r*(As···I) = 3.591 Å [153]. The large difference is attributed to the presence of secondary C–S···I chalcogen-bonded interactions formed by the same I^–^ anion when in attractive engagement with two nearest neighbor S atoms (∠C–S···I = 151.4° and 124.9°; *r*(S···I) = 3.618 and 3.943 Å). It is not that the As···I contact cannot be longer. The crystal structure of tetraisopropylcyclopentadienyl arsenic(III) diiodide (TipCpAsI_2_) is an example of where a pair of As···I contacts uniquely hold the building blocks together in the crystal (Figure 7a,b) [154]. The As···I intermolecular distance is 4.031 Å with an angle of approach of the electrophile (∠C–As···I) of 143.4°. The significant nonlinearity in the pnictogen bonds in this system is because the negative site on I around the As–I covalent bond is affected by secondary H···I hydrogen bonds. Nevertheless, the pnictogen-bonding environment is very similar between building blocks responsible for the bromide derivative (TipCpAsBr_2_, CSD ref. code NUDVIR) [154]. However, in this case, the As···Br intermolecular distance is 3.878 Å and ∠C–As···Br = 135.1°, showing nonlinearity of pnictogen bonding in the bromide system as well.

**Figure 8:**
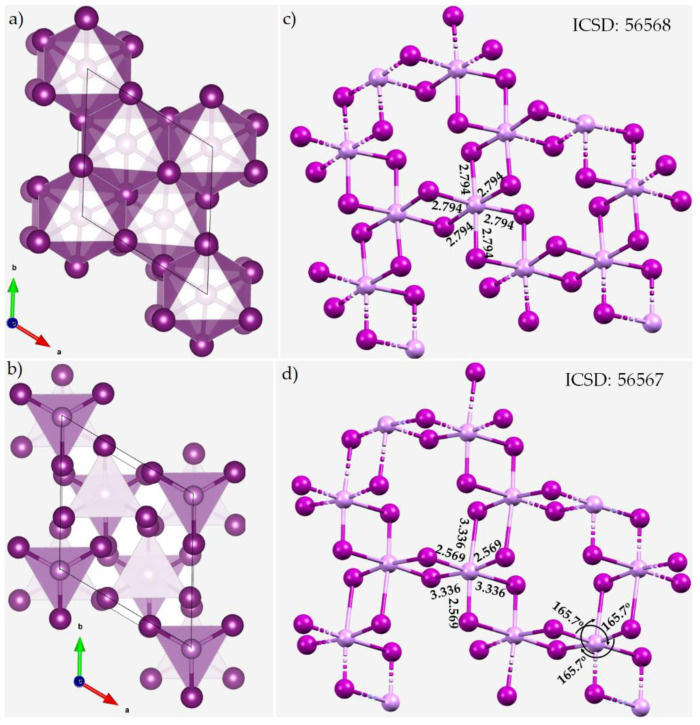
(**a**,**b**) The polyhedral model and ball-and-stick models of the crystal of AsI_3_. (**c**,**d**) The ball-and-stick models of a layer of the corresponding crystals, respectively. Selected As–I bond lengths and angles are shown in Å and degree, respectively. ICSD code is shown in each case. Atoms and bonds are shown as balls and sticks in atom color: As—faint-purple; I—purple.

In addition to forming a network of halogen and pnictogen bonding between discrete molecular entities in the solid state, AsI_3_ also forms layered crystals (Figure 8). In this case, the As is octahedral in a given layer and each layer is linked with another layer through I···I halogen bonding. The packing between the AsI_3_ molecules is such that each arsenic site along the three I–As bond extensions in a given molecular entity is coupled to the negative lateral portions localized on I around the As–I bond extensions on an interacting molecule.

Hsueh et al. used angle-dispersive powder X-ray diffraction and Raman spectroscopy to examine the structural, vibrational, and electronic properties of a prototypical family of quasi-molecular layered solids of the type PnI_3_ (Pn = As, Sb, Bi) under compression [157]. They found that an unusual non-monotonic variation of the symmetric Pn−I stretching frequency could be unambiguously attributed to the formation of intermolecular bonds and that compression results in a sequence of transitions from a hexagonal molecular structure, to a hexagonal layered structure, then to a monoclinic structure. The ICSD database contains two structures of the AsI_3_ system with *R-*$\bar{3}$ space group symmetry. These layered structures are shown in Figure 8.

The octahedral structure of AsI_3_, with six equivalent As–I bonds in each layer, is shown in Figure 8a,c, while the trigonal pyramidal structure with three short and three long As–I bonds is shown in Figure 8b,d. Because of the marked differences in the As–I bonds in the latter system (2.569 Å vs. 3.336 Å), we attribute the three longer As–I bonds to As···I contacts, which have the characteristics of Type-IIa pnictogen bonds.

We have also used As···I bond distances in the range of 2.6–4.5 Å and ∠R–As···I in the range of 140–180° to search for the As···I close contacts in the crystals deposited to CSD. This provided a total of 59 hits (131 close contacts), with two false results (*vide supra*). The CSD search also included (some) halogen bonds in the list of 131 close contacts in which covalently bonded I forms directional I···As interactions in those crystals. For example, in the crystal structure of (C_3_H_9_As,C_6_F_5_I) (Figure 7c) [155], *r*(I···As) = 3.367Å and ∠C–I···As = 171.5° and was unavoidably included in the list of 131 close contacts. The same crystal also contains As···I close contacts, with *r*(As···I) = 4.317 Å and ∠C–As···I = 166.5°. The latter are nothing other than arsenic bonds given that the axial portion of covalently bonded As interacts with the lateral portion of I.

In the case of the crystal structure of (C_3_H_9_AsI_2_) (CSD ref. ZODJOR, Figure 7d [156]), close contacts certainly occur, but the (I)I···As contact is no longer a halogen bond given that it is already involved in the formation of a covalent coordinate bond with As in As(CH_3_)_3_ (*r*(I···As) = 2.271 Å and ∠C–I···As = 180.0°); hence, in this molecular entity, As is four-coordinate. In addition, covalently bound As in As(CH_3_)_3_ also donates three arsenic bonds to the three nearest neighbor I atoms that are longer (*r*(As···I) = 4.234 Å and ∠C–As···I = 179.6° each) and accepts one I···As halogen bond (*r*(I···As) = 3.696 Å and ∠C–I···As = 180.0°), showing that As in As(CH_3_)_3_ is effectively eight-coordinate (Figure 7d).

The formation of such close contacts in both crystals is supported by the MESP model shown in Figure 7e. As can be seen in Figure 7b (left), there are three positive σ-holes on the three C–As bond extensions in As(CH_3_)_3_ (*V_S,max_* associated with each is 9.5 kcal mol^−1^); each faces the negative lateral portion of the I atom in C_6_F_5_I (Figure 7d). The lateral portion of the As atom along the extension of the *C_3v_* axis is strongly negative (*V_S,min_* = –23.5 kcal mol^−1^), a feature that was not observed in arsenic trihalides (cf. Figure 1) in which the former also faces the positive σ-hole observed on the I atom along the C–I bond extension. In addition, we also found that the methyl carbon does not feature a σ-hole on the As–C bond extensions as is usually observed in molecules likeCH_4_ [158,159]; instead, the σ-hole area features a negative potential (*V_S,min_* = –2.2 kcal mol^−1^) and As is positive on the opposite site of the *C_3v_* axis (Figure 7e, right).

These long arsenic bonds noted above were also found in other crystals, such as, for example, 5-iodo-5H-benzo[b]arsindole (*r*(As···I) = 4.325 Å and ∠C–As···I = 174.2°, CSD ref. BAVFOW [160]), dihydroxybis(methyl)arsonium bis(dimethylarsinic acid) tri-iodide (*r*(As···I) = 4.485 Å and ∠O–As···I = 156.8°, CSD ref. BUBZEE [161]), and tetramethylarsonium iodide (*r*(As···I) = 4.287/4.395 Å and ∠C–As···I = 180/174.6°, CSD ref: YOKSOG [162]). In some of these crystal systems, H···I hydrogen bonds between the building blocks play a crucial role in the formation of the solid-state structure and this drives the formation of the arsenic bonds.

From Figure 7f,g, we found that the peak of the normal distribution associated with 130 As···I contacts (all false contacts) center at 162° and 3.80 Å for the bond angle and bond distance, respectively, and there are no As···I contacts < 3.1 Å as was the case with the As···Br close contacts (see Figure 5g,h). Most As···I close contacts occur between 3.6–4.5 Å, with the contact angles fairly evenly distributed between 155 and 180°. As with As···Cl and As···Br close contacts, we found that many As···I close contacts in the representative crystals do not strictly obey the “less than the sum of the vdW radii” criterion..

### 5.3. The As···S Arsenic Bonds

The electron density distribution in covalently bound sulfur, S, in molecules is also anisotropic [69,163]. The electrostatic surface of S is therefore positive and negative, respectively, along and around a covalently bonded sulfur atom.

A search of the CSD for crystals containing As···S close contacts led to 148 hits (306 instances of close contacts) when the intermolecular bond distance As···S and bond angle ∠R–As···S were constrained to the ranges of 2.6–4.3 Å and 140–180°, respectively. Of these 148 results, we found 46 crystals that contained 99 false close contacts. The majority these false contacts occur between S in CF_3_SO_3_^−^ and As, or between As in AsF_6_^−^ and S. A visual inspection of each and every supposed result was required to eliminate these false results. The inspection revealed that a great number of As···S close contacts in the remaining 102 crystals (207 contacts) may be regarded as arsenic bonds, although a few could be attributed to chalcogen bonds.

**Figure 9:**
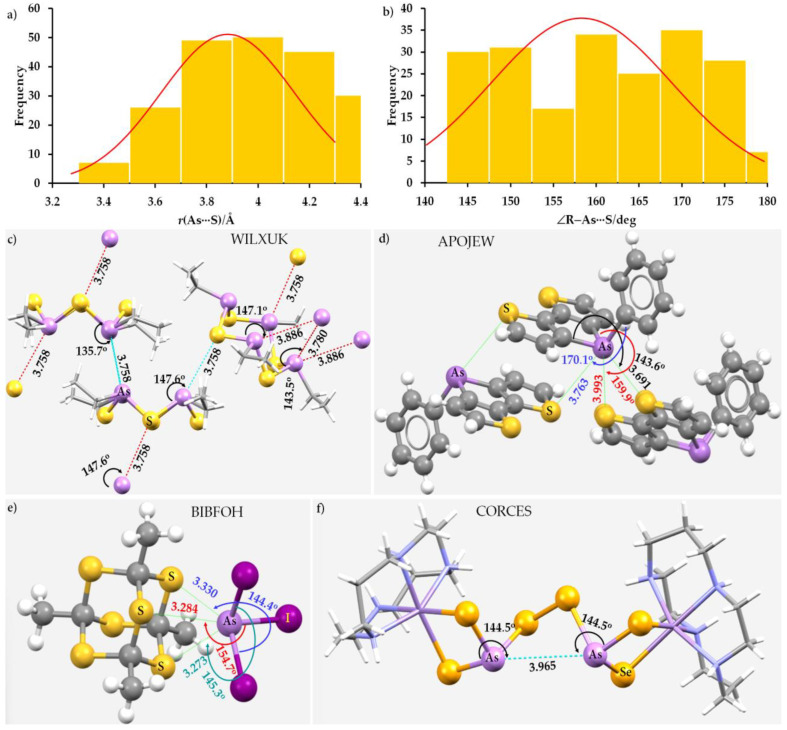
(**a**,**b**) Histograms showing the distribution of 207 As···S close contacts in 102 crystals. The As···S intermolecular distance As···S (and ∠R–As···S) in the range of 2.6–4.3 Å (145–180°) was used as a criterion during the CSD search, where R represents any atom of the periodic table. The ball-and-stick model of the crystal structure of (**c**) cyclo-(C_2_H_5_AsS)_4_, (**d**) C_14_H_9_AsS_2_, and (**e**) [C_8_H_12_S_6_][AsI_3_], showing the possibility of As···S and/or As···As intermolecular interactions between the building blocks of these crystals wherever feasible. (**f**) The type of As···As intramolecular contact in the crystal of [Mn(cyclam)]_2_[(AsSe_2_)_2_(μ-Se_2_)]. Selected bond lengths and angles are shown in Å and degrees, respectively. Hanging contacts are represented by dotted lines in red. CSD references in capital letters are shown.

Figure 9a,b show the histogram plots of the 207 As···S close contacts, with all false results eliminated. As can be seen, the As···S bond distances fall largely in the range of 3.6–4.2 Å, with a peak of the normal distribution around 3.88 Å. Similarly, ∠R–As···S are broadly distributed in the range of 145–175°, which is a signature of the Type-II topology of bonding (Scheme 1). The data in the histogram plots show that many crystals possess As···S contacts that are longer than the sum of the vdW radii of As and S atomic basins, 3.77 Å. Examples include tri-iodoarsine tris(octathiocane) (3(S_8_)·AsI_3_, CSD ref. YIWNEA [164]), (2RS,4RS)-5-(2-Phenyl-1,3,2-dithiarsinan-4-yl)pentanoic acid (C_14_H_19_AsO_2_S_2_, CSD ref: NIDKAM [165]), (arsenotrithionito)-bis(ethane-1,2-diamine)-chromium(III) (C_4_H_16_AsCrN_4_S_3_, CSD ref. EYAJOG [166]), and catena-[bis(m_3_-arsorotrithioito)-bis(propane-1,2-diamine)-tri-manganese] ((C_6_H_20_As_2_Mn_3_N_4_S_6_)_n_, CSD ref: TACDEJ [167]). These have (*r*(As···S) = 3.974 Å and ∠I–As···S = 157.8°), (*r*(As···S) = 4.143 Å and ∠S–As···S = 170.8°), (*r*(As···S) = 4.196 Å and ∠S–As···S = 159.2°), and (*r*(As···S) = 4.115 Å and ∠S–As···S = 156.3°), respectively.

In the following set of examples, we illustrate how the lateral negative portion of S in some molecules is capable of donating electron density to the acidic region on bonded As in interacting molecular entities in some crystalline materials.

As shown in Figure 9c, the molecular units in the cycloarsathiane, cyclo-(C_2_H_5_AsS)_4_, Ref. [168], are linked to each other through As···S and As···As contacts, as well as through H···S hydrogen bonds. The first have the characteristics of Type-IIb, nonlinear pnictogen bonds. The S–As···As contacts are somewhat longer than the As···S contacts, as expected since the vdW radius of As is marginally much larger than that of S (1.89 Å vs. 1.88 Å). The significant nonlinearity in the pnictogen bonds is mainly due to the presence of H···S hydrogen-bonded interactions. Although the directionality of the As···As and As···S interactions was judged based on the S–As···As and S–As···S angles shown in Figure 9c, the intermolecular distance associated with each of the two equivalent As···As pnictogen bonds is slightly longer than twice the vdW radius of As, 3.76 Å. A similar pattern was found in the silver complex of the same cycloarsathiane, [Ag(cyclo-(C_2_H_5_AsS)_4_]_2_]CF_3_SO_3_, as well as in the structure of its co-crystallization with SbBr_3_, [cyclo-(C_2_H_5_AsS)_4_]·2SbBr_3_ [168]. In the case of the structure where two Mn(II) cyclam complexes are bridged by [(AsSe_2_)_2_(μ-Se_2_)]^4−^ [169], seen in Figure 9f, the As···As contact is slightly longer, intramolecular, and nonlinear (r(As···As) = 3.965 Å; ∠Se–As···As = 144.5°).

For crystals C_14_H_9_AsS_2_ [170] and [C_8_H_12_S_6_][AsI_3_] [171], shown in Figure 9d,e, respectively, both Type-IIa and Type-IIb arsenic bonds occur. In the former, there are two Type-IIa (∠C–As···As > 150°) and one Type-IIb (140° < ∠C–As···As < 150°) contacts, and in the latter we observed one Type-IIa (∠C–As···As > 150°) and two Type-IIb (140° < ∠C–As···As < 150°) contacts. They are much stronger in [C_8_H_12_S_6_][AsI_3_] than those found in the crystal of C_14_H_9_AsS_2_ and cyclo-(C_2_H_5_AsS)_4_, [168].

**Figure 10:**
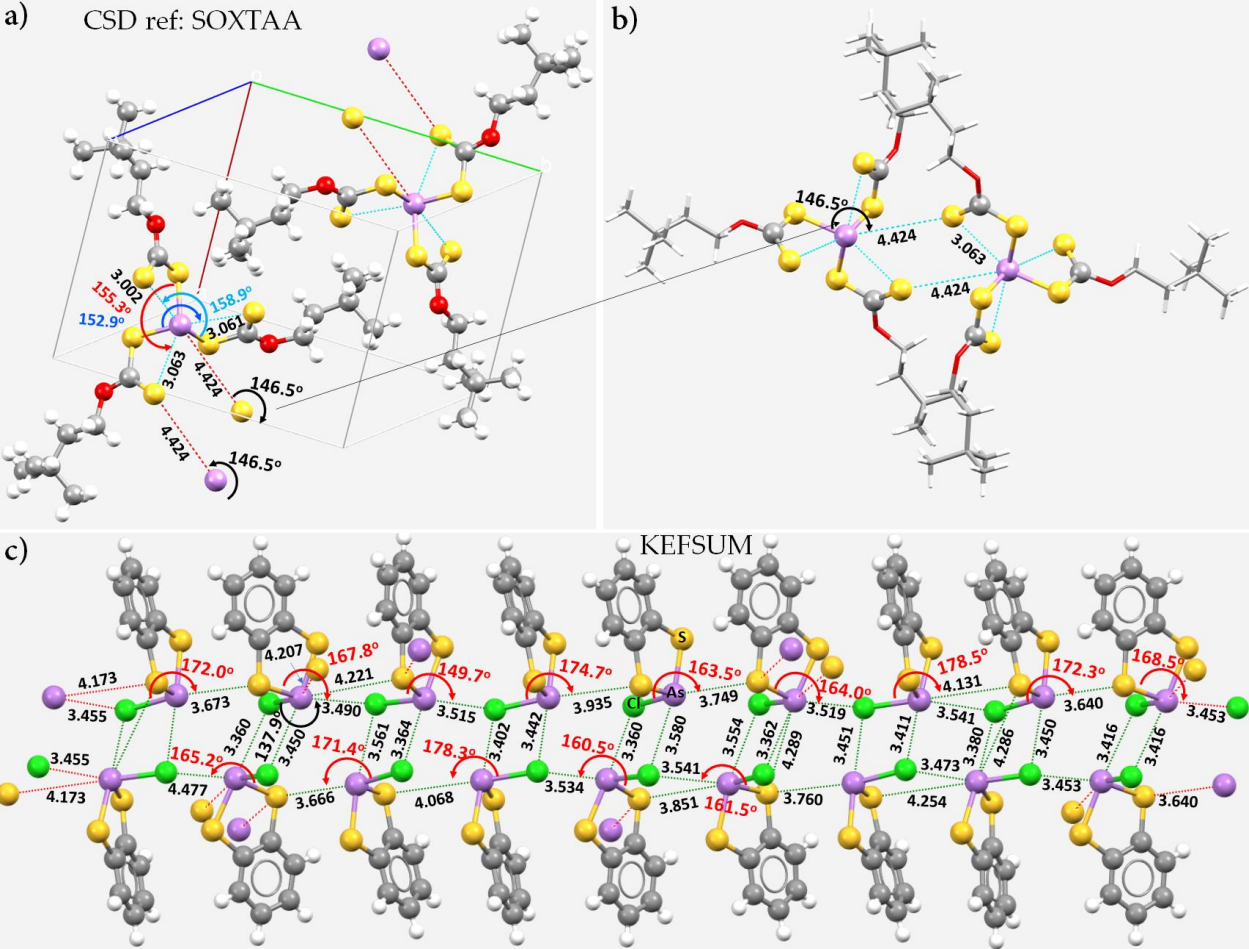
(**a**) The unit-cell of the crystal of As(S_2_COCH_2_CH_2_CMe_3_)_3_, showing the local topology of As···S inter- and intramolecular interactions. (**b**) Illustration of As···S inter- and intramolecular interactions between a pair of building blocks, with long-ranged As···S contacts (dotted lines in cyan). (**c**) The ball-and-stick model of the repeating units of the building blocks along the titled *b*-direction in the crystal of C_6_H_4_S_2_AsCl (2-chloro-2H-1,3,2-benzodithiarsole), showing a network of As···S and As···Cl arsenic bonds responsible for the crystal. Hanging contacts represented by dotted lines in red. Bond lengths and angles are shown in Å and degree, respectively.

As(S_2_COCH_2_CH_2_CMe_3_)_3_, seen in Figure 10, is another example of a crystal system in which both intramolecular and intermolecular pnictogen bonds, in addition to H···O and H···S hydrogen bonds, occur [172]. As^3+^ forms three short bonds and three long bonds with the negative sites on covalently bound S in three thiocarbonate moieties and is thus pseudo-six-coordinate. The bond lengths of the three short As–S bonds are 2.300, 2.295 and 2.294 Å. These are genuine coordinate bonds between As^3+^ and RS^-^ and have polar covalent characteristics. The remaining three As···S bonds are substantially longer, with bond distances of 3.061, 3.063, and 3.002 Å. These are intramolecular arsenic bonds. They are directional and the ∠S–As···O corresponding to these three contacts are 152.9°, 155.3°, and 158.9°. The crystal system may also feature intermolecular As···S pnictogen bonds that are by far longer. There are two such bonds formed between a pair of building blocks (Figure 10b). The intermolecular distances (and angles of interaction) corresponding to each of these is 4.424 (146.5°). These results show that intramolecular interactions may be relatively more directional than intermolecular interactions.

Tran et al. [173] reported the synthesis of the benzo-fused dithia-chloro-arsole derivative C_6_H_4_S_2_AsCl (2-chloro-2H-1,3,2-benzodithiarsole) that crystallizes in the triclinic space group *P*$\bar{1}$ with 17 molecules in the asymmetric unit. They also reported a tolyl derivative, MeC_6_H_3_S_2_AsCl, that is polymorphic, with the α-phase crystallizing in the monoclinic space group *P*2_1_/*c* with a single molecule in the asymmetric unit, and with the β-phase adopting a triclinic structure with two molecules in the asymmetric unit. When they reacted with LiN(SiMe_3_)_2_ in a 3:1 mole ratio, the dithia-chloro-arsole derivatives resulted in a unique paddlewheel structure (MeC_6_H_4_S_2_As)_3_N. The structure of C_6_H_4_S_2_AsCl is shown in Figure 10c. In this structure, the bound As site in each C_6_H_4_S_2_AsCl unit is either seven- or eight-coordinate; five of them are significantly longer than the remaining three. The former are characteristic of arsenic bonds that appear as As···S and As···Cl close contacts and are not equivalent. Although Type-IIa contacts are very common, Type-IIb contacts are not very rare (not shown). In addition, As···As Type-Ia and -Ib arsenic contacts are evident throughout the crystal structure, with *r*(As···As) = 3.875–4.011 Å and S–As···As ≈ 118–137.7°.

**Figure 11:**
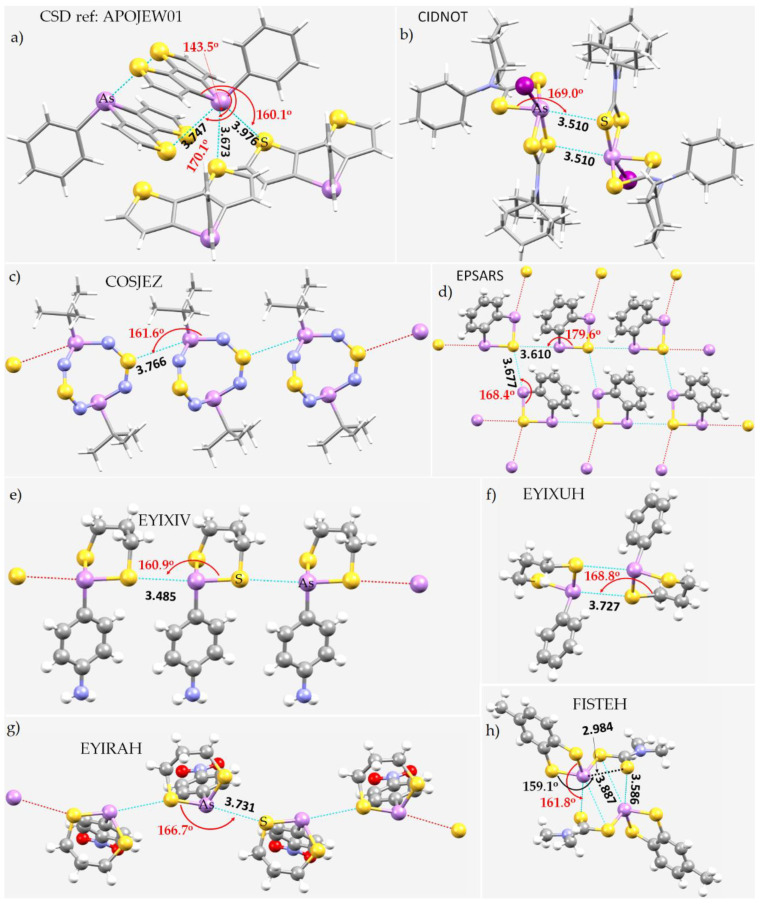
Examples showing the attractive engagement of sulfur as a Lewis base in some molecules with the acidic part of As in interacting molecular entities, contributing to the stability of the crystalline material. (**a**) 4-phenyl-arsolo [3,2-*b*:4,5-*b*’]dithiophene (C_14_H_9_AsS_2_) [174]; (**b**) ((cyc-hex)_2_(NCS_2_)_2_AsI, iodo-bis(*N,N’*-bis(cyclohexyl)dithiocarbamato)arsenic(III) [177]; (**c**) (tBuAs_2_)_2_(N_2_S), 3,7-di-t-butyl-dithia-tetraazadiarsocine [178]; (**d**) (Ph_2_As_2_)S, 5,10-epithio-5,10-dihydroarsanthrene [179]; (**e**) (NH_2_Ph)As(S_2_(CH_2_)_3_), 4-(1,3,2-dithiarsinan-2-yl)aniline [180]; (**f**) PhAs(S_2_(CH_2_)_3_) 2-phenyl-1,3,2-dithiarsinane [180]; (**g**) (NO_2_Ph)As(S_2_(CH_2_)_3_), 2-(4-nitrophenyl)-1,3,2-dithiarsinane [180]; (**h**) Me_2_NC(S)SAs(S_2_(3*S*,4*S*-1-MePh)), (N,N-dimethyldithiocarbamato)(toluene-3,4-dithiolato)arsenic(III) [175]. Bond lengths and angles are shown in Å and degree, respectively. Primary and secondary intermolecular interactions are not shown. Selected atom types are marked in (**a**,**b**,**e**,**g**).

A further set of eight structures that feature As···S contacts between the molecular building blocks that help shape the crystal structure are shown in Figure 11a–h. The longest and shortest of these are observed in the crystal structures of a polymer containing fused dithieno [3,2-*b:*2′,3′-*d*]arsole units (4-phenyl-4H-arsolo [3,2-*b*:4,5-*b*’]dithiophene) [174] and an As^3+^ complex of a dialkylthiocarbamate ((*N,N*-dimethyldithiocarbamato)(toluene-3,4-dithiolato)As) [175], respectively. The first has *r*(As···S) = 3.976 Å and ∠C–As···S = 160.1° (Figure 11a), and the second, *r*(As···S) = 2.984 Å and ∠C–As···S = 159.1° (Figure 11h). As is evident from Figure 11, the angle of approach for the formation of an arsenic bond varies between 159° and 180° and depends on the nature of the skeletal framework of the interacting partners since they determine whether the positive site on As will be along or off the bond axis. The eight-membered As_2_N_4_S_2_ ring of the C_8_H_18_As_2_N_4_S_2_ molecule found in the crystal shown in Figure 11c has an As···As intramolecular bond distance of 3.683 Å, with the two ∠C–As···As of 162.0° and 165.3°. Although these two angles feature a Type-IIa topology, the As···As intramolecular interaction may be regarded as Type-III. The occurrence of this is not surprising since the surface regions of the two interacting As sites have a similar charge polarity but a different charge density, and this allows them to sustain a mutual interaction. Type-III topologies of bonding between similar atomic basins of different molecular entities have been discussed elsewhere [60,176].

### 5.4. The As···N, As···N(π) and As···C(π) Arsenic Bonds

**Figure 12:**
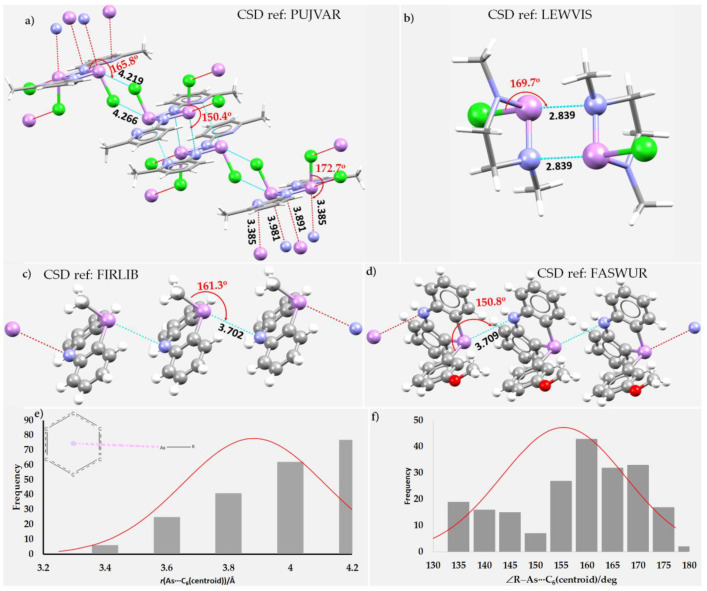
Examples showing As···N pnictogen bonds in the crystals of (**a**) ((6-Me(C_4_H_3_N)NAsCl)_2_ bis(*μ*_2_-(6-methyl-2-pyridyl)amido)-syn-dichloro-di-arsenic(III) [181]; (**b**) (CH_2_N(CH_3_))_2_AsCl, 2-chloro-1,3-dimethyl-1,3-diaza-2-arsolidine [183]; (**c**) (Ph_2_NH)AsCH_3_, 5-methyl-5,10-dihydrophenarsazine [184]; and (**d**) (Ph_2_NH)As(3-MeOPh), 5-(3-methoxyphenyl)-5,10-dihydrophenarsazine [185]. Selected atom type is shown in (**b**–**d**). Selected bond lengths *r* and bond angles ∠ are shown in Å and degree, respectively. Hanging contacts are shown by dotted red lines. (**e**,**f**) Histograms showing the nature of distribution of 211 arsenic contacts to the centroid of an aromatic ring bond distance *r* and bond angle ∠ found for 158 crystals. The skeletal diagram in the insert in (**e**) was used for CSD search of crystals with the centroid of the aromatic ring to covalently bonded As intermolecular distances (angles) in the ranges of 2.6–4.2 Å and 130–180°, respectively, and R in the insert represents any element of the periodic table.

The formation of arsenic bonds where N acts as the Lewis base is quite common and a number of such structures have been deposited into the CSD. Four examples are shown in Figure 12. The geminal N-centered arsenic(III) amide [[2-(6-Me)pyridyl]NAsCl]_2_ is one [181]. The intermolecular interactions evident in this material are similar to those also found in [[2-(6-Me)C_5_H_3_N]N(AsCl_2_)_2_] [181] and [2-(6-Me)C_5_H_3_N]NSiMe_3_(AsCl_2_)] [182].

From the geometric features shown in Figure 12a, it is likely that As···N(π) and As···Cl play a critical role in stabilizing the crystal structure. Other interactions, such as Cl···Cl Type-I halogen bonds, H···Cl hydrogen bonds, π···π-staking interactions, and Type-II As···C(π) intermolecular pnictogen bonds contribute to the packing arrangement. The As···C(π) bonds are not only markedly longer but somewhat less directional than the As···N(π) bonds. In the case of the crystal structure of (CH_2_N(CH_3_))_2_AsCl (Figure 12b), the As···N bond distances are substantially shorter than those observed in the structures shown in Figure 12a,c,d. It is clear that the bond length and the strength of the As···N bonds depend on the nature, size, and steric crowding of interacting molecular entities in the crystal. 1,2,4-Diaza-arsole is another such system (CSD ref. HELPOD [186]) where As···N(π) interactions between the interacting molecular units determine the geometry of the crystal.

We searched CSD for instances of covalently bound arsenic interacting with the centroid of an aromatic ring bond. The distance *r* and bond angle *A*, *A* = ∠R–As···arene(centroid), were constrained to the ranges of 2.6–3.7 Å and 140–180°, respectively. This found 38 hits that included 56 close contacts. The peak distribution occurred at bond distance *r* (20 instances) in the range of 3.56–3.69 Å and *A* between 156 and 166°. We carefully examined these 38 crystals and no outlier was found, meaning that no false contacts between the interacting units were found. When the distance and angular ranges were constrained to the ranges of 2.6–4.0 Å and 150–180°, respectively, the CSD search found 100 hits that comprised 117 instances of close contacts in the crystals containing arsenic bonds and the peak distribution of *r* (36) and *A* (36) was found in the ranges of 3.81–3.95 Å and 154–158°, respectively; no false results were found.

When the above ranges were constrained to 2.6–4.0 Å and 140–180°, our search led to 114 hits that comprised a total of 139 geometric instances with the maximum occurrence of *r* (36) and *A* (36) in the ranges of 3.90–3.95 Å and 158–160°, respectively, and with no false results. When the ranges were constrained to 2.6–4.2 Å and 130–180°, the search found 158 results that comprised 211 geometric instances with a few outliers. We did not continue this search beyond this range since the occurrence of false contacts was expected to increase significantly. The extension of the bond angle beyond the above range will result in the spurious identification of arsenic-centered pnictogen-bonded interactions in many crystals.

Figure 12e,f show the histograms containing the 211 geometric instances, illustrating the nature of the natural distribution of bond distances and bond angles in the crystals. From these results, it is quite apparent that the π density of arene moieties plays a significant role in the development of arsenic bonds, and that the peak of the normal distribution of the bond distance and bond angle curves is around 3.9 Å and 155°, respectively, and that there are no As···arene(centroid) contacts found below 3.2 Å. While all possibilities, such as N, S, and O atoms, in arene moieties of different sizes were not explored, we believe that inclusion of all such possibilities will lead to an increase in the number of instances of arsenic bonds in structures deposited into the CSD.

### 5.5. Other As-Centered Arsenic Bonds (As···O, As···X, As···N and As···As)

**Figure 13:**
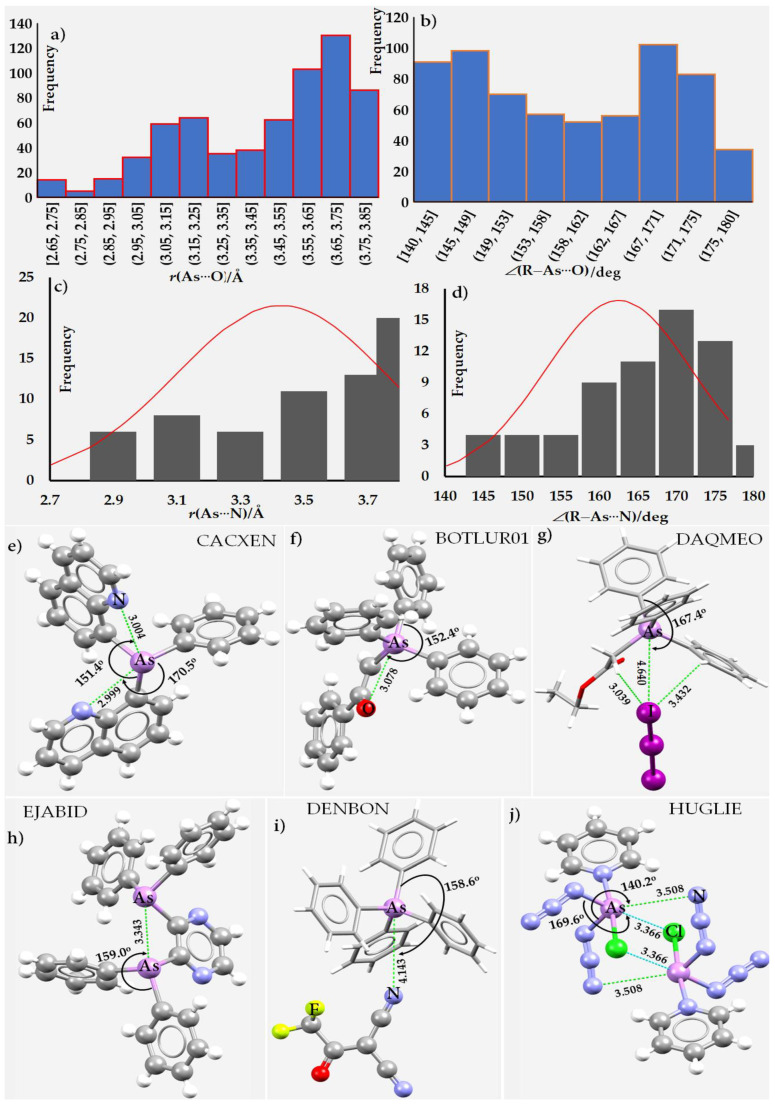
(**a**,**b**) Histograms showing the distribution of 643 As···O contacts and ∠R–As···O in 317 crystals emerged from a CSD search (see text for details). (**c**,**d**) Histograms showing the distribution of 64 As···N contacts and ∠R–As···N in 43 crystals emerged from a CSD search, with the normal distribution curve in red. Illustration of inter- and intramolecular arsenic bonds in some crystals catalogued in the CSD, including: (**e**) 8,8′-(phenylarsanediyl)diquinoline [193]; (**f**) 1-phenyl-2-(triphenyl-arsanylidene)ethan-1-one [194]; (**g**) triphenyl(ethoxycarbonylmethyl)arsonium tri-iodide [196]; (**h**) 2,3-bis(diphenylarsino)pyrazine [195]; (**i**) tetraphenylarsonium trifluoroacetylide-dicyanomethanide [197]; (**j**) diazido-chloro-pyridyl-arsenic(III) [198]. Selected bond lengths and angles are shown in Å and degree, respectively. In case of (**c**,**e**), both the capped stick and ball-and-stick models are presented for clarity.

To demonstrate the very wide occurrence of As-centered noncovalent interactions, we have examined a variety of crystal systems and searched the CSD for As···O contacts. The intermolecular bond distance and bond angle were limited to 2.6–3.5 Å and 160–180°, respectively. This found 97 hits containing 224 geometrical instances. However, when the ranges were expanded to 2.6–3.8 Å and 150–180° (2.6–3.8 Å and 140–180°), the number of crystal structures satisfying the criteria increased to 218 (317), with 436 (643) geometric instances. The latter comprising 643 As···O results is shown in Figure 13a,b; several outliers may be likely as we did not investigate the geometric details of the 317 crystals.

Although several distorted structures were unavoidably included in the 643 hits, there were only a few crystals identified with linear As···O close contacts. One such undistorted system was the crystal of tetramethylarsonium superoxide (ammonia solvate), C_4_H_12_As^+^O_2_^–^·2(NH_3_), CSD ref: PEVXUK [187], in which, *r*(As···O) = 3.302 Å and ∠C–As···O = 179.8°. Arsonoacetic acid is another crystal (CSD ref: DIVQAC [188]) that has *r*(As···O) = 3.369 Å and ∠O–As···O = 179.6°. In the case of ammonium dihydrogen arsorate arsoric acid (CSD ref. HORWET [189]), there are a variety of As···O contacts; one of them has an *r*(As···O) = 3.476 Å and ∠O–As···O = 178.2° and occurs between two negative sites (Type-III). Similarly, one of the significantly distorted crystals identified was that of bis(tetra-*n*-butylammonium) octakis(*m*-hydroxo)-octadecakis(*m*-pyrazolato)-pentadeca-copper diarsenate (bromobenzene nitrobenzene solvate), [C_54_H_62_Cu_15_N_36_O_8_^4+^, 2(C_16_H_36_ N^+^), 2(AsO_4_^3−^)]·2(C_6_H_5_Br)·2(C_6_H_5_NO_2_), CSD ref LUDZOB [190], with *r*(O–As···O) = 3.320 Å and ∠O–As···O = 179.3°. The arsenic-centered interaction in the first system is a genuine Type-IIa arsenic-bonded interaction, whereas that in the second is not an arsenic bond since As in AsO_4_^3−^ carries a negative charge, and O in C_54_H_62_Cu_15_N_36_O_8_^4+^ carries a positive charge; this is therefore a chalcogen-bonded interaction.

Our CSD search showed that, in an appreciable number of crystals, the As···O distance is longer than the sum of the vdW radii of As and O atomic basins, 3.38 Å. These include, to list just three, crystals such as C_28_H_36_As_4_Cl_2_O_16_Pd (*r*(As···O) = 3.773 Å and ∠Pd–As···O = 171.6°, CSD ref: JIZMIP [191]), C_8_H_11_AsBBr_2_NO_5_WPd (*r*(As···O) = 3.786 Å and ∠Br–As···O = 159.2°, CSD ref: JOJZEO [192]), and 10-phenoxarsine sulfide (*r*(As···O) = 3.666 Å and ∠S–As···O = 150.1°, CSD ref: POXARS [184]).

Searching for the presence of As···N close contacts in crystals with As···N intermolecular bond distances and ∠R–As···N bond angles in the range of 2.6–3.8 Å and 160–180°, respectively, yielded 58 hits with 81 close contacts. When the geometric constraint was limited to 2.6–3.8 Å and 150–180° (2.6–3.8 Å and 140–180°) for As···N and ∠R–As···N, respectively, the number of results in the CSD search was 100 (113), corresponding to 136 (187) close contacts. We examined the 187 close contacts in 113 results and found that a large number of these contacts (123) were false results. There were 64 close contacts in 43 crystals that are considered to be genuine As···N contacts. Although the search criteria were met, the CSD search overlooked the presence of ammine hydrogen atoms, among others, that were involved in hydrogen bonding with O, S, or N atoms of interacting molecular entities, thereby causing the emergence of a large number of false As···N contacts. However, our observations indicate that many As···N arsenic bonds are present between the molecular building blocks in various crystal lattices, contributing to their skeletal stability. Figure 13c,d represent the histogram plots of the 64 possible close contacts, indicating that As···N close contacts are also populated around 3.7 Å and the peak of the normal distribution of bond distances and bond angles occurred around 3.4 and 162.5°, respectively.

**Figure 14:**
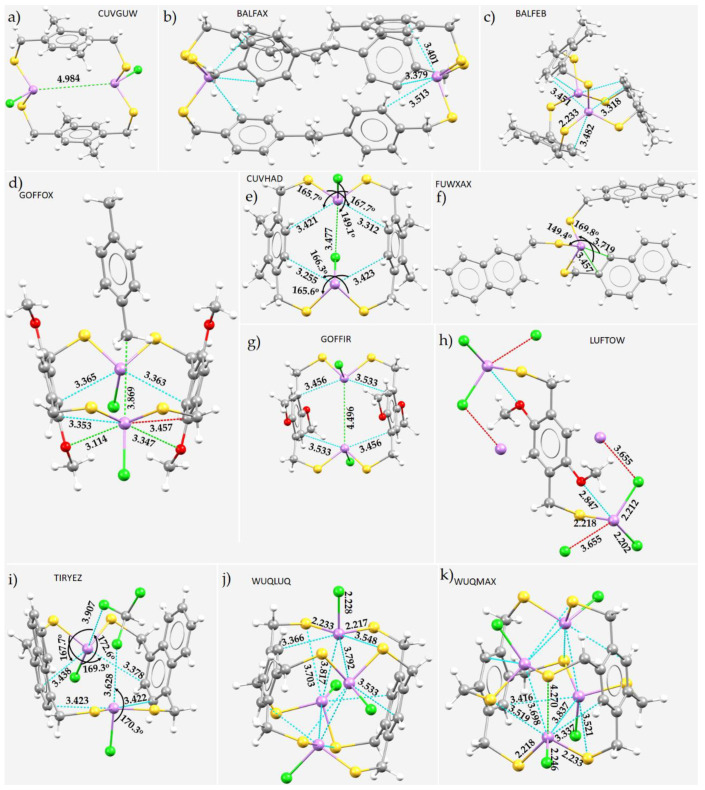
The nature of As-centered intramolecular (in some cases intermolecular) bonding modes in some crystals. (**a**) (*μ*_2_-(2,5-dimethylbenzene-1,4-diyl)dimethanethiolato)_2_(AsCl)_2_ [128]; (**b**) tris(*μ*_2_-(ethane-1,2-diyldibenzene-4,1-diyl)dimethanethiolato)As_2_ [200]; (**c**) tris(μ_2_-(2,3-dimethylbenzene-1,4-diyl)dimethanethiolato)As_2_ [200]; (**d**) syn-bis(μ_2_-1,4-dimethoxy-2,5-bis(mercaptomethyl)-benzene)(AsCl)_2_ hemikis(p-xylene) clathrate [201]; (**e**) bis(*μ*_2_-(2,3-dimethylbenzene-1,4-diyl)dimethanethiolato)(AsCl)_2_ [128]; (**f**) tris(2-naphthylmethyl) arsenotrithioite [128]; (**g**) anti-bis(*μ*_2_-1,4-dimethoxy-2,5-bis(mercaptomethyl)benzene)(AsCl)_2_ [201]; (**h**) (2,5-dimethoxy-1,4-phenylene)-bis(methylene)(AsCl_2_)_2_ [202]; (**i**) syn-bis(*μ*_2_-1,4-bis(mercaptomethyl)naphthalene)(AsCl)_2_ [203]; (**j**) [As_4_L_2_Cl_4_], L = 1,2,4,5,-tetrakis(mercaptomethyl)benzene [199]; (**k**) [As_4_L_2_Cl_4_]·[As_2_LCl_2_], L = 1,2,4,5,-tetrakis(mercaptomethyl)benzene [199]. Selected bond lengths and angles are shown in Å and degree, respectively. Atom type is shown in (**d**).

Further examples in which intermolecular and intramolecular arsenic bonds are evident in crystals are shown in Figure 13 and Figure 14. The angular features shown in Figure 13c,d and f indicate that the As···N, As···O and As···As intramolecular contacts are quasi-linear in the phenyl diquinoline complex (CSD ref. code CACXEN) [193], the keto arsenic ylid (BOTLUR01) [194], and the diarsine complex (EJABID) [195]. The contacts are shorter than the sum of the vdW radii of the respective atomic basins.

Three other crystal systems in which intermolecular arsenic bonds are evident are shown in Figure 13g,i,j. The longest of these is in the crystal of the triodide salt, DAQMEO [196], with *r*(As···I) = 4.640 Å. Although it is significantly longer than the sum of the vdW radii of As and I (3.92 Å), it is directional, with ∠C–As···I = 167.4°. A very similar feature occurs in the two crystals shown in Figure 13i (DENBON) [197] and Figure 13j (HUGLIE) [198]; the As···N intermolecular distances are longer than the sum of the vdW radii of As and N, 3.54 Å (*r_vdW_* (As) = 1.88 Å and *r_vdW_* (N) = 1.66 Å). In the case of DAQMEO [196] (Figure 13g) and DENBON [197] (Figure 13i), the Lewis bases forming the As···I and As···N arsenic bonds are also involved in H···I and H···N hydrogen bonds with the arene moiety (not shown) and these are probably the primary interactions that cause the formation of secondary pnictogen bonds.

The putative As···As interactions in Figure 14a (CUVGUW) and Figure 14g (GOFFIR) may be too long to be considered arsenic bonds. However, the dotted lines between As and electron-donating fragments shown in all the other molecular systems of Figure 14 may be regarded as such. Some of them are nonlinear and others are quasi-linear (cf. Figure 14e,f,i). The latter are along the extension of the S–As and Cl–As bonds, and are all significantly longer than the coordinate covalent bonds (cf. Figure 14h). We also identified two intramolecular As···As contacts (*r*(As···As) = 3.817 and 3.792 Å) in the crystal structure of [As_4_L_2_Cl_4_], L = 1,2,4,5,-tetrakis(mercaptomethyl)benzene [199], (Figure 14j), which are marginally longer than twice the vdW radius of As, 3.76 Å. They are expected to be weaker than the As···S, As···Cl, As···O, and As···Cπ arsenic bonds evident in the other systems. In the case of the crystal [As4L2Cl4]·[As2LCl2], L = 1,2,4,5,-tetrakis(mercaptomethyl)benzene shown in Figure 14k, the arsenic bond lengths are 3.837 and 3.698 Å. Simultaneously, covalently bound As is engaged with other donors, forming As···S and As···Cπ intramolecular contacts. Among the examples depicted in Figure 14, it seems that the As···O contacts are shortest in LUFTOW (Figure 14h); hence, they are stronger than the inter- and intramolecular contacts found in the other systems shown.

A CSD search of structures with As···As intramolecular distance in the range of 3.0–3.7 Å and ∠R–As···As (R = any element) in the range of 160.0°–180.0° found 97 hits; many of the identified structures adopt a cage-like geometry. The highest frequency of the As···As bond (36) and ∠R–As···As (28) was found in the ranges of 3.35–3.90 Å and 160–161°. Although there were many false contacts identified, some crystals display As···As intramolecular interactions similar to those shown in Figure 11c and Figure 13d. These include S(NAsPh_2_)_2_ (CSD ref. COSJID [178]) and (CSD ref. COSJID01 [204]), bis(dimethylamido)-bis(*N*-phenyl-As,As-bis(2,4,6-trimethylphenyl)arsinous amidato)-hafnium (CSD ref. KORCAY) [205], and (*μ*_2_-calix(4)arene-25,26,27,28-tetraolato)-bis((dimethylamino)-arsenic) (CSD ref. ZALGUO) [206]. The *r*(As···As) bond distances in these systems are 3.379, 3.362, 3.519, and 3.343 Å, respectively, and the values of ∠R–As···As are 168.0°, 168.72°, 169.74°, and 173.15°, respectively. In the case of [C_24_H_20_P]^2+^[As_4_Se_6_]^2−^ (CSD refs. WADNOE [207] and SIDQUR [208]), the As···As intramolecular interaction occurs within the framework of the [As_4_Se_6_]^2−^ dianion itself, with r(As···As) = 3.637/3.618 Å and ∠Se–As···As = 162.5°/163.1°, respectively. In the bis(di-*n*-propylammonium) hexothiotetra-arsenate (CSD ref. NEMJUK [209]), [C_6_H_16_N]^2+^[As_4_S_6_]^2−^, and [C_13_H_28_N_2_]^2+^[As_4_S_6_]^2−^ (CSD ref. KEWZOE [210]) crystals, the As···As contact in the [As_4_Se_6_]^2−^ dianion has *r*(As···As) = 3.517 and 3.506 Å and ∠Se–As···As = 163.7° and 164.6°, respectively. In the case of 5,5′-([1,1′-biphenyl]-2-diyl)di(5H-benzo[b]arsindole) (CSD ref. HAQNUL [211]), C_36_H_24_As_2_, and (C_30_ H_22_ As_4_ Mn_2_ N_6_ S_8_)_n_ (CSD ref. CONDOZ [212]) crystals, *r*(As···As) = 3.248 and 3.590 Å, respectively, and ∠C–As···As = 178.7° (177.6°) and 167.88°, respectively. These are signatures of Type-III interactions (see Scheme 1), which may not be described by the MESP model.

**Figure 15:**
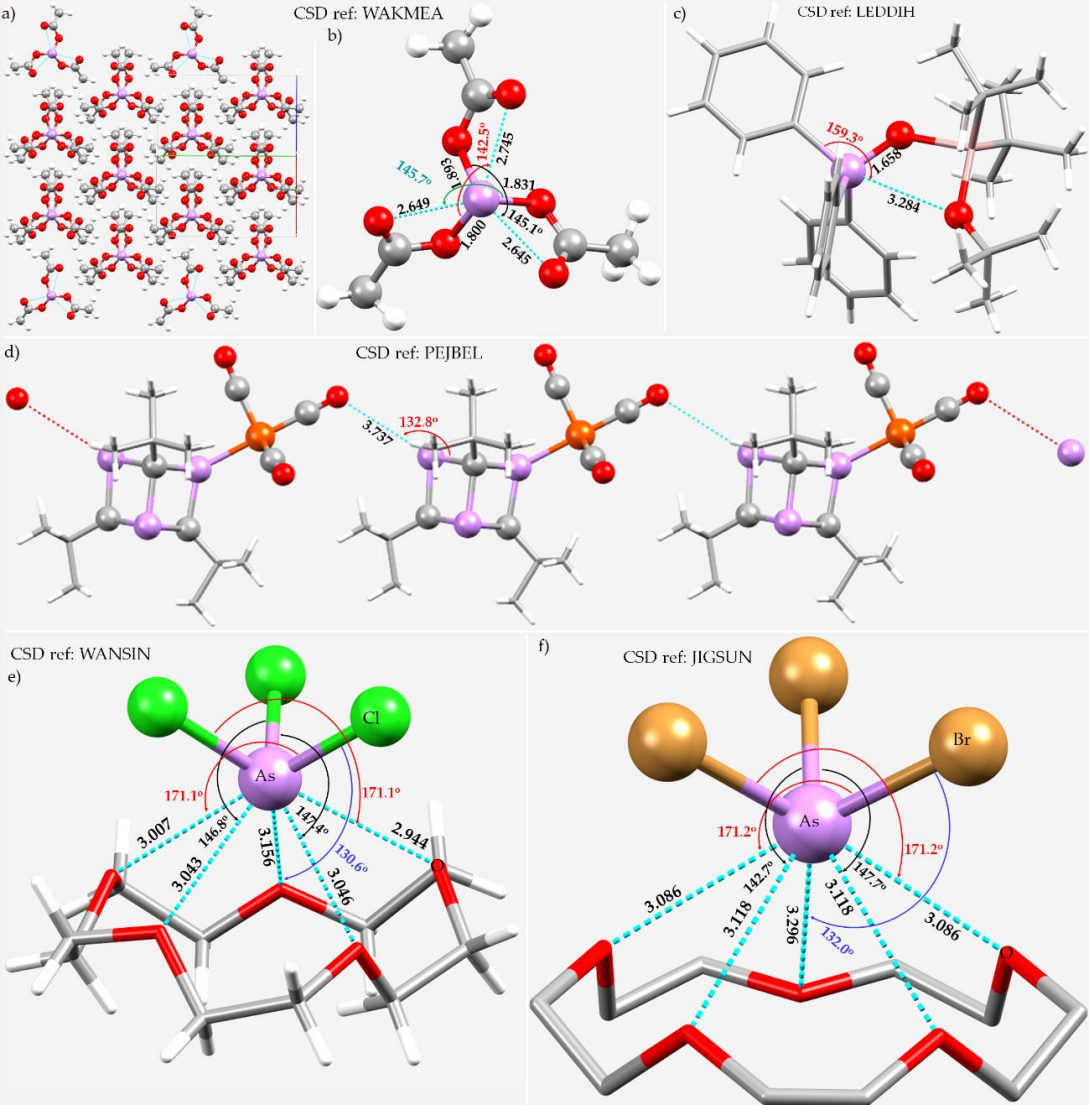
Illustration of the (**a**) 2 × 2 × 2 supercell and (**b**) molecular analogues of the crystal of tris(acetato)arsenic [213]. Also shown are molecular/oligomeric versions of the crystals of (**c**) (μ_2_-oxo)-(di-t-butyl)-(t-butoxy)-triphenyl-arsenic-gallium, (tBu)_2_Ga(OtBu)(O=AsPh_3_) [214]; (**d**) tetra-arsa-tetra-t-butylcubane-tetracarbonyl-iron(0), (As_3_(C-tBu)_3_·Fe(CO)4 [215]; (**e**) (15-crown-5)-trichloro-arsenic [216]; and (**f**) (15-crown-5)-tribromoarsenic [217]. Atom type is marked in (**e**,**f**). Selected bond distances and bond angles are in Å and degree, respectively. H atoms are not shown in (**f**).

While the vdW radius of O is markedly shorter than that of other electron density donors considered above (viz. N, Cl, F, Br, I and As), it is clear that O can form noncovalent interactions of varying length as an acceptor of pnictogen bonds. This is shown in Figure 15a–d. The first two show very short and very long As···O intramolecular pnictogen bonds in the crystals of As(OCOCH_3_)_3_ (WAKMEA) [213] (Figure 15a,b) and (^t^Bu)_2_Ga(O^t^Bu)(O = AsPh_3_) (LEDDIH) [214] (Figure 15c), respectively. They are substantially longer than the As–O covalent bonds of 1.800–1.893 and 1.658 Å, respectively, found in the two crystal structures. In the case of the complex between the cubane ligand (As_3_(C-^t^Bu)_3_ and Fe(CO)_4_ [215], PEJBEL (Figure 15d), the As···O contact, is intermolecular by nature, and is significantly nonlinear.

The crystal structures of 15-crown-5 ether and arsenic trihalide(s) are shown in Figure 15e (WANSIN) [216] and 15f (JIGSUN) [217]. In them, the As^3+^ center in AsX_3_ (X = Cl, Br) is penta-furcated, donating five arsenic-centered pnictogen bonds to the guest 15-crown-5. In these, the As···O intermolecular contact distances are substantially longer than the As–X bond distances (e.g., 3.70 Å in Figure 15f), and hence can be regarded as arsenic-centered pnictogen bonds. Of the five As···O contacts in tribromo-(15-crown-5)-arsenic, two are short and equivalent (viz. 3.086 Å each in Figure 15f) and display strong directional behavior (∠Br–As···O = 171.2° each). Two other contacts are slightly longer and are equivalent (3.118 Å) and are bent (∠Br–As···O = 147.7°). The fifth As···O contact is the longest and is significantly nonlinear (∠Br–As···O = 132.0°) and is comparable with that of ∠C–As···O = 132.8° in PEJBEL (Figure 15d). A similar structural feature is evident in the crystal of (15-crown-5)-trichloro-arsenic, WANSIN (Figure 15e). The intermolecular distances of all the five contacts are less than the sum of the vdW radii of As and O atomic basins, 3.43 Å. This provides evidence of the ability of a covalently bound arsenic to be accommodated in a cage-like structure. It also resembles a similar chemical bonding topology observed in late-transition metal cations when they find themselves in close proximity to 15-crown-5 [218] and 18-azacrown-6 [219].

### 5.6. As···Se and As···Te Arsenic Bonds with Tellurium and Selenium As Electron Density Donors

**Figure 16:**
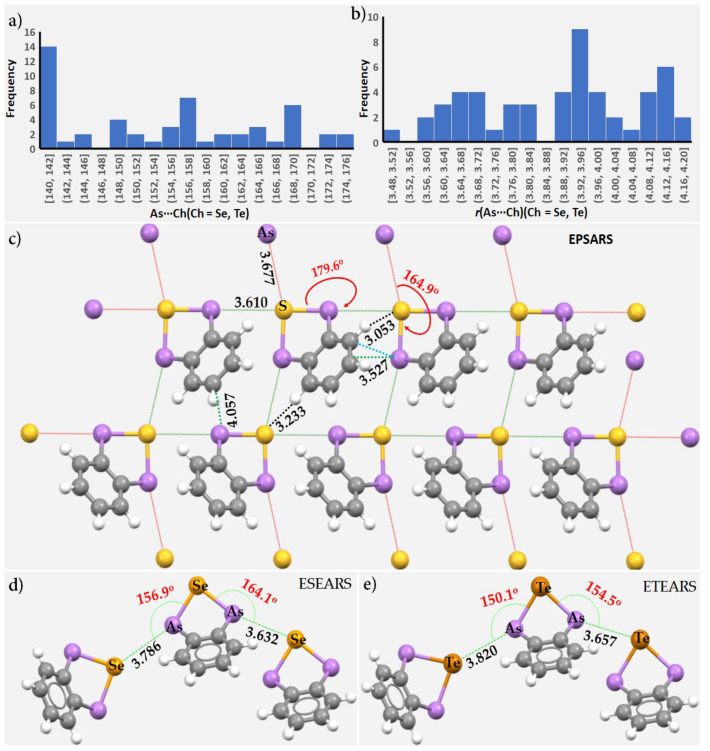
(**a**,**b**) Histograms showing the distribution of ∠R–As···Ch and As···Ch (Ch = Se, Te) contacts, respectively, in 22 crystals emerged from a CSD search (see text for details), where R refers to any element of the periodic table. Illustration of As···Ch (Ch = S, Se, Te) arsenic bonds in the ball-and-stick models of C_12_H_8_As_2_Ch crystals: (**c**) C_12_H_8_As_2_S; (**d**) C_12_H_8_As_2_Se; (**e**) C_12_H_8_As_2_Te. Selected bond lengths and angles are shown in Å and degree, respectively, and CSD reference is shown in each case. Atom types involved in the formation of arsenic bonds are labeled in each case. Contacts are shown as dotted lines in different colors.

A CSD search of structures with the As···Ch (Ch = Se, Te) intramolecular distance in the range of 2.9–4.0 Å and ∠R–As···As (R = any element) in the range of 160.0°–180.0° as geometric constraints found seven hits. When these two ranges changed to 2.8–4.2 Å and 140.0°–180.0°, our search found 29 hits. We found no false results with the first search criteria, but the second found seven, which we discarded. Some of them, for example, include structures with the intermolecular distances and angles between a six-coordinate octahedral As ion in a molecular entity and the Ch atom in the neighboring entity, as in the crystal structures of [[S_4_Te_4_]^2+^[AsF_6_]_2_^–^]·SO_2_] (CSD ref. GEJMOX) [220] and [N_2_Se_3_]^2+^[AsF_6_]^-^_2_ (CSD ref. KAZBOC) [221]. As shown in the histograms in Figure 16a,b, the ∠R–As···As and As···Ch (Ch = Se, Te) for the 22 crystals were observed to be in the ranges of 140.0°–176° and 3.48–4.2 Å, respectively, indicating that the As···Se and As···Te contacts identified were either quasi-linear or nonlinear. The plots also show that the largest angle and distance occurrences were around 140.0°–142.0° and 385–4.15 Å, respectively. When S was included, the CSD search provided a large number of results (*vide supra*). This suggests that the relatively more electronegative chalcogen atoms readily serve as an electron density donor for the electrophilic region on the covalently bound As, i.e., the frequency is in the order S >> Se > Te. Even so, the occurrence of outliers increases significantly and this is not very straightforward to handle (*vide supra*).

A few crystal systems that feature As···Ch (Ch = Se, Te) arsenic bonds are shown in Figure 16c–e. All of them show the way that the As···Ch arsenic-bonded contacts connect the building blocks in the crystal lattice. These include the 5,10-epithio-, 5,10-episeleno-, and 5,10-epitelluro-5,10-dihydroarsanthren, C_12_H_8_As_2_Ch (Ch = S, Se, Te), series [179,222]. A notable geometric feature of these crystals is the As···Ch (Ch = S, Se, Te) contacts, even though the molecular geometry is quite similar for all the three compounds that adopt a ‘butterfly’ conformation around the plane of the As-Ch-As atoms and the molecules of all three compounds show non-crystallographic symmetry of 2 mm around an axis bisecting the As-Ch-As bond angle. The As···Ch (Ch = S, Se, Te) contacts have deviated from linearity in all three crystals; As···S is more linear than As···Se and As···Te. This is understandable since Te is more electropositive than Se and S; hence, the As center in the partner molecule adjusts its position to maximize its attractive noncovalent interaction with Te and Se more so than the interaction formed with S. Although the possibility of these interactions were not mentioned in the original studies [179,222], they are very likely to be present given that the As···Ch intermolecular distance is less than the respective vdW radii sum of As and S (3.77 Å), As and Se (3.70 Å), or As and Te (3.87 Å), where *r*_vdW_ (As) = 1.88 Å, *r*_vdW_ (S) = 1.89 Å, and *r*_vdW_ (Se) = 1.82 Å; *r*_vdW_ (Te) = 1.99 Å [82].

The geometric stability of the C_12_H_8_As_2_Ch (Ch = S, Se, Te) crystals is not just the result of the As···Ch intermolecular contacts. There are two other equivalent As···C_π_ (arene) pnictogen-bonded interactions between the building blocks that are equally strong (*r*(As···C_π_) = 3.527 Å for each in Figure 16c, 3.543 Å for each in Figure 16d, and 3.657 Å for each in Figure 16e). The H···Ch hydrogen bonds between arene’s C–H and the Ch fragment of the interacting moiety are feasible in these crystals and are stronger in C_12_H_8_As_2_S than in C_12_H_8_As_2_Ch (Ch = Se, Te). These three types of interactions are collectively responsible for the development of the 1D chain-like architecture of the crystal along the crystallographic *c*-direction (see Figure 16c). The 1D chains are physically linked with the nearest 1D chains via long and less directional As···Ch intermolecular contacts, as well as via As···C_π_ (arene) and H···Ch hydrogen-bonded contacts (see Figure 16c). For instance, the long *r*(As···Ch) values responsible for the 2D architecture of the C_12_H_8_As_2_S, C_12_H_8_As_2_Se, and C_12_H_8_As_2_Te crystals are 3.677, 3.786, and 3.820 Å, respectively. These bonding features signify that As in C_12_H_8_As_2_Ch (Ch = S, Se, Te) is cable of forming three σ-hole interactions and that the As ion may be considered to be pseudo six-coordinate.

**Figure 17:**
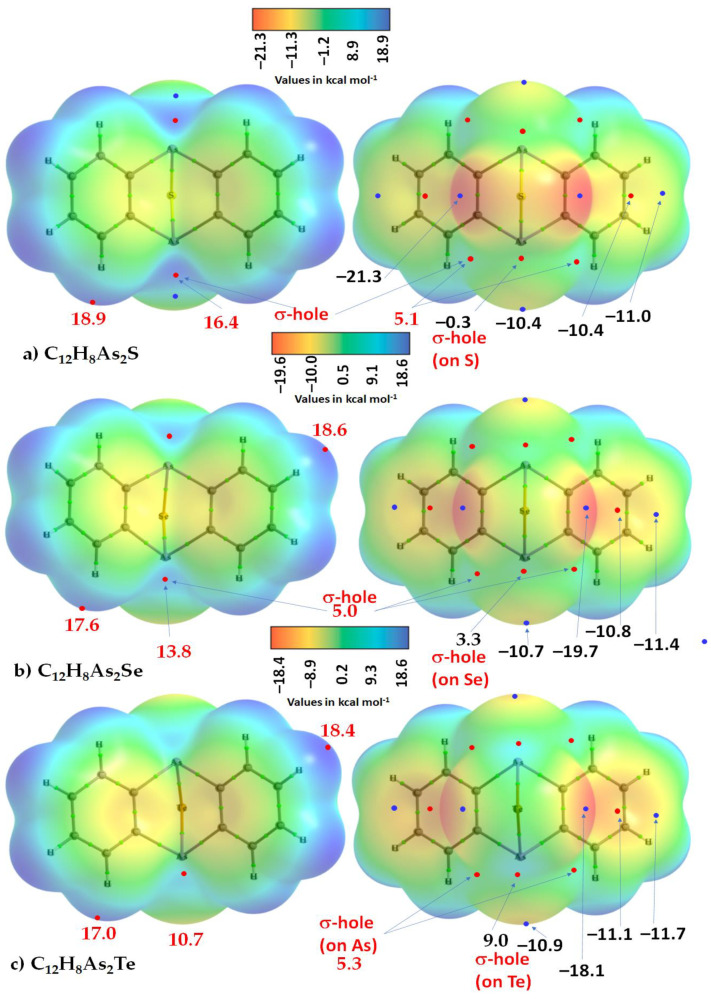
0.001 a.u. isodensity mapped electrostatic potential on the surfaces of C_12_H_8_As_2_Ch (Ch = S, Se, Te) molecules, obtained with ωb97XD/Aug-CC-pVTZ (-PP): (**a**) C_12_H_8_As_2_S; (**b**) C_12_H_8_As_2_Se; (**c**) C_12_H_8_As_2_Te. The local most minimum and maximum of electrostatic potentials represented by *V_S,min_* and *V_S,max_* are marked by tiny circles in blue and red, respectively. Color bar is depicted for each case. Two views are shown for each case: (left) the bridging chalcogen atom orienting opposite to the viewer; (right) the bridging chalcogen atom orienting towards the viewer.

That the covalently bound As in isolated C_12_H_8_As_2_Ch (Ch = S, Se, Te) monomers is positive can be inferred from the MESP graphs shown in Figure 17. Each covalently bonded As site in each of the three molecules has three σ-holes. Its strength on As along the Ch–As bond extension is stronger, whereas that along the two C–As bond extensions are equivalent and weaker. Along the chalcogen series, its strength follows the order S–As (16.4 kcal mol^−1^) > Se–As (13.8 kcal mol^−1^) > Te–As (13.8 kcal mol^−1^), indicating that S in the molecule has relatively more of an electron-density-withdrawing capability than Se and Te; this is in accord with the order of their electronegativities (S > Se > Te) [102,103].

The covalently bound Ch atom in all the three molecules has two σ-holes. They are equivalent and negative in C_12_H_8_As_2_S along the two As–S bond extensions (*V_S,max_* = –0.3 kcal mol^−1^) and are equivalent and positive in C_12_H_8_As_2_Se and C_12_H_8_As_2_Te (*V_S,max_* values for the corresponding systems are 3.3 and 9.0 kcal mol^−1^, respectively). It is also notable that the surface of each As in C_12_H_8_As_2_Ch is not entirely positive since the local minimum of potential on its surface (lateral portions) carries a negative sign. For instance, the *V_S,min_* value is –10.4, –10.7, and –10.9 kcal mol^−1^ on As for C_12_H_8_As_2_S, C_12_H_8_As_2_Se, and C_12_H_8_As_2_Te, respectively. Among the σ-holes identified, that on H along the C–H bond extensions was found to be the strongest. By contrast, the charge density was found to be anisotropic on the surfaces of bonded Ch sites. It becomes more electrophilic as one passes from S through Se to Te, and the lone-pair density resides opposite to the As–Ch bonding direction. The latter are displayed as red regions on Ch in Figure 17a–c (see graphs on right), and that the magnitude of the negative *V_S,min_* value follows the order S (–21.3 kcal mol^−1^) > Se (–19.7 kcal mol^−1^) > Te (–18.1 kcal mol^−1^). The anisotropic nature of the Ch atoms in C_12_H_8_As_2_Ch (Ch = S, Se, Te) explains why the arsenic bonds are relatively more directional in C_12_H_8_As_2_S crystal than in C_12_H_8_As_2_Ch (Ch = Se, Te).

**Figure 18:**
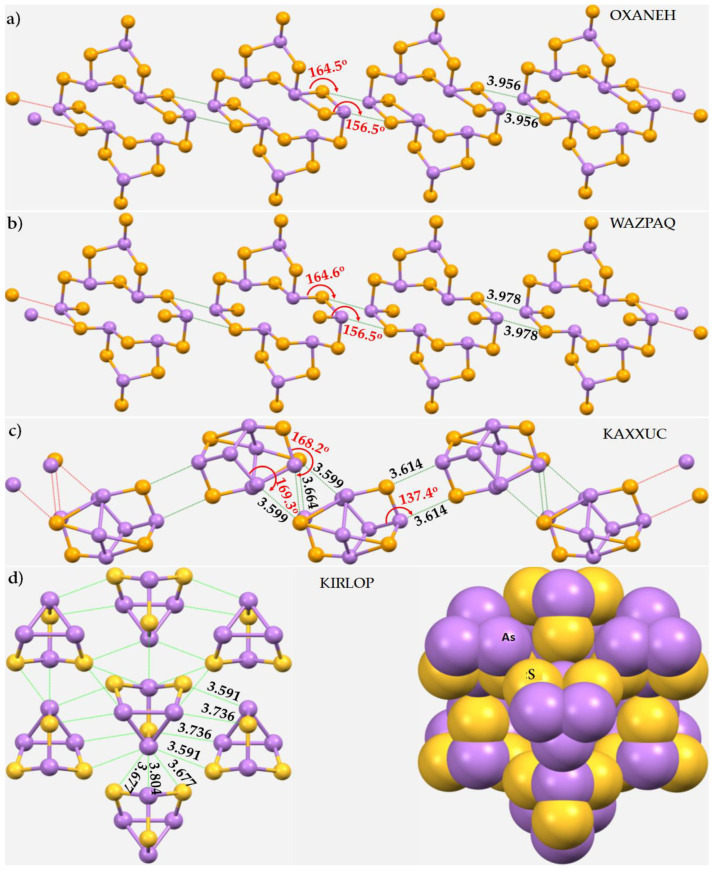
Illustration of specific As···Se contacts in some crystals: (**a**) bis(tris(1,10-phenanthroline)-cobalt(II)) tetradeca-selenido-octa-arsenate (2[C_36_H_24_CoN_6_)^2+^]·[As_8_Se_14_]^4–^) [223]; (**b**) tris(1,10-phenanthroline)-zinc(II) tetradecaselenido-octa-arsenic (2[C_36_H_24_N_6_Zn]^2+^·[As_8_Se_14_^4–^]) [224]; (**c**) tetraphenylphosphonium hepta-arsenic-tetraselenate ([C_24_H_20_P]^+^[As_7_Se_4_]^–^) [225]; (**d**) 3,5,7-trithia-1,2,4,6-tetraarsatricyclo [2.2.1.0^2,6^]heptane (As_4_S_3_) [227]. The cations are deleted in (**a**–**c**) for clarity. While the ball-and-stick representations are shown for all occasions, a space-filling model is included for the structure shown in (**d**) to clarify the nature of atom-atom overlapping between the interacting units responsible for the crystal. Selected bond distances and bond angles are in Å and degree, respectively. Atom type is shown in (**d**) and the CSD reference in capital letters is marked for each case.

The crystals shown in Figure 18a–c are ion-pair adducts. The As···Se contacts are 3.978 and 3.956 Å in bis(tris(1,10-phenanthroline)-cobalt(II)) tetradeca-selenido-octa-arsenate (2[C_36_H_24_CoN_6_)^2+^][As_8_Se_14_]^4–^) [223] and tris(1,10-phenanthroline)-zinc(II) tetradecaselenido-octa-arsenic (2[C_36_H_24_N_6_Zn]^2+^[As_8_Se_14_^4–^]) [224], respectively. They are longer than the vdW radii of As and Se (3.70 Å). They are directional and are significantly nonlinear (∠Se–As···Se = 156.5°). As for these systems, we also observed attraction between the [As_7_Se_4_]^–^ anions, forming As···Se contacts in the ion-pair adduct [C_24_H_20_P]^+^[As_7_Se_4_]^–^ (CSD ref: KAXXUC) [225]; see Figure 18c. In all these three crystal systems, it is apparent that the intermolecular bonding between the interacting units follows a Type-III topology (Scheme 1). It is difficult at this stage to verify whether they genuinely occur in the crystal, or whether they appear as a forced consequence of the [C_24_H_20_P]^+^ cation that brings the anions together in close proximity. In any case, the nature of attraction between the interacting anions may be comparable with that reported for the B_24_I_18_]^2–^ moiety. It was shown using multiscale first-principles calculations [226] that two interacting negatively charged [B_12_I_9_]^–^ monoanions not only attract each other, in defiance of Coulomb’s law, but also the energy barrier at 400 K was small enough that these two moieties combine to form a stable [B_24_I_18_]^2–^ moiety, in agreement with the experimental observation of the “spontaneous” formation of [B_24_I_18_]^2–^ in an ion trap. The results of a simple model based on electrostatics enabled the authors to demonstrate that the unusual attraction between the monoanions is due to the competition between the attractive dipole–dipole interaction caused by the aspherical shape of the particle and the repulsive interaction between the like charges. There are other reports that demonstrate that two positive sites, or two negative sites, can attract each other when placed in close proximity.

In case of crystalline As_4_S_3_ [227], seen in Figure 18d, the network of intermolecular arsenic bonding is quite complex. The As center in each cluster molecule links with the surrounding neighbors via As···S arsenic bonds, S···As chalcogen bonds, as well as via As···As contacts. For instance, the As···S (∠As–As···S) contact distances (angles) that are 3.677 (112.8°) are Type-IIb, the S···As (∠As–S···As) contact distances (angles) that are 3.591 (147.1°) are Type-IIb, and the As···S (∠As–As···As) contact distances (angles) that are 3.736 (167.3°) are Type-IIa. Similarly, the As···As (∠As–As···As) contact distances (angles) are 3.804 (147.0°) or 3.945 Å (127.4°). These show that Type-IIa and Type-IIb interaction topologies of bonding occur simultaneously between the interacting units. Evidently, the competition between various bonding interactions between the As_4_S_3_ cage molecules provide stability to the overall crystal of the system. It was shown in [227] that Ag(As_4_S_3_)_2_[Al(OR^F^)_4_] displays a paddlewheel structure with interconnecting As_4_S_3_ ligands and is the first-known example of a homoleptic metal (As_4_S_3_) complex.

**Figure 19:**
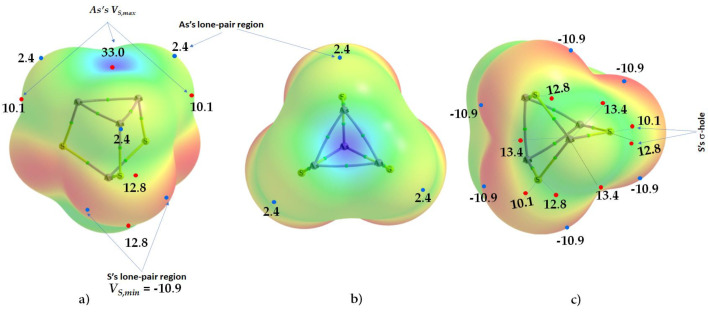
(**a**–**c**) Three different orientations of ωB97XD/cc-pVTZ level 0.001 a.u. isodensity envelope mapped electrostatic potential on the surface of molecular As_4_S_3_. *V_S,min_* and *V_S,max_* values (marked by tiny circles in blue and red, respectively) are shown in kcal mol^−1^, which represent the local most minimum and the local most maximum of potential, respectively.

The intermolecular bonding between the As_4_S_3_ cage molecules described above may be consistent with the MESP model shown in Figure 19. As can be seen from Figure 19a, the triangular face formed by the As_3_ motif is entirely positive. The bluish hole-like region is sufficiently electron density deficient, with *V_S,max_* = 33.0 kcal mol^−1^. The supposed lone-pair regions on bonded As are completely neutralized; hence, *V_S,min_* values on the lateral portion of As are positive (*V_S,min_* = 2.4 kcal mol^−1^ on each As, Figure 19a). Moreover, the three σ-holes on the surface of S-bonded As (the one bonded to three S atoms) are equivalent, with *V_S,max_* = 13.4 kcal mol^−1^ (Figure 19c). On the other hand, a pair of σ-holes along the two As–S bond extensions is inequivalent (*V_S,max_* 12.8 and 10.1 kcal mol^−1^; see Figure 19c), whereas the lateral sites on each of the three S atoms in As_4_S_3_ are very negative and are described by the reddish regions (*V_S,min_* = −10.9 kcal mol^−1^ on each S; see Figure 19a,c).

### 5.7. The Crystals of Arsenic

**Figure 20:**
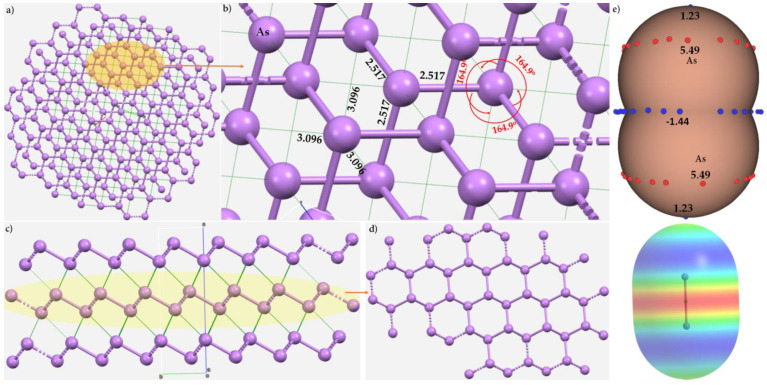
(**a**) The 2D structure of the low temperature (4.2 K) phase of arsenic crystal (space group: *R-3mH* (166)) reported in 1969, showing the network of As-centered pnictogen-bonding interactions between the monolayers in a tri-layer system (ICSD ref: 16516 [228]); (**b**) the local topology of As···As bonding interactions between two monolayers; (**c**) the zig-zag nature of the As···As connectivity in the crystal; (**d**) top view of the monolayer; (**e**) space-filling models showing the 0.001 a.u. mapped extrema of potential on the electrostatic surface of molecular As_2_ obtained with MP2/cc-pVTZ: (top) van der Waals surface of As_2_ with extrema of potential and (bottom) the actual MESP of As_2_ with color bar (extrema of potential not shown).

As does not always behave as an electrophilic center. As already shown above, there is crystallographic evidence that it can act as an electron density donor (cf. Figure 7e) when in molecular entities. What follows is a brief discussion on the structure of As [228], Figure 20a, which is a 2D-layered crystal and is different to the two other known forms, orthorhombic and rhombohedral (metallic) arsenic [229]. The As···As noncovalent links around each As site are displayed as dotted lines, and the As–As covalent coordinate bonds as sticks in atom color. As can be seen in Figure 20b, the former links are significantly longer than the latter (2.517 Å vs. 3.096 Å), thus providing As a pseudo-octahedral local environment within the bilayer arrangement (Figure 20c). This octahedral topology around As is not feasible within the monolayer called arsenene [230,231], as is understood from Figure 20d, where each As is locally trigonal, with the connectivity between As sites forming a honeycomb-like hexagonal structure within the monolayer.

The three long As···As contacts, feasible only in the bilayer system, are directional. For instance, ∠As–As···As for each of these three contacts is 164.9° (Figure 19b), appearing along the extension of the As–As bond. While these may be assumed to be appearing as the result of σ-hole interactions, that is not the case. We confirmed this by examining the electrostatic potential of an As_2_ molecule. As shown in Figure 20e, the electrostatic surface of the As_2_ molecule comprises both positive and negative regions described by the positive and negative potentials, respectively. Contrary to what has been found for diatomic molecules such as HX (X = F, Cl, Br, I) and X_2_ (X = F, Cl, Br, I), the As atom in As_2_ features two positive *V_S,min_* regions along the As–As bond extensions (*V_S,min_* = 1.23 kcal mol^−1^ each). This is less positive than the equatorial sites of the same atom described by a belt of positive electrostatic potential (*V_S,min_* = 5.49 kcal mol^−1^). The surface of the molecule features a belt of negative positive potential around the bonding region (*V_S,min_* = −1.44 kcal mol^−1^). This picture emerged from the MP2/cc-pVTZ level of theory; we cross-validated the nature of the electrostatic potential using two other basis sets, Aug-cc-pVTZ and def2-DZVPPD. Although a basis set has some effect on the magnitude of the 0.001 a.u. isoelectron density mapped potential, this did not change the observations given above; the three types of potential calculated around the As–As bond critical point region, along and around the As–As bond extensions, were 2.34, 5.88, and −1.71 kcal mol^−1^, respectively, using Aug-cc-pVTZ; and 1.30, 5.26, and −1.90 kcal mol^−1^, respectively, using def2-DZVPPD. We also used MP2/aug-cc-pVTZ to compute the MESP of the molecule by fixing the As–As bond distance at 2.517 Å as in the layered crystal (Figure 20b). The potential along and around the As–As bond extension were 5.46 and 5.5 kcal mol^−1^, respectively, and the molecules have a *V_S,min_* value of −2.5 kcal mol^−1^ around the bonding region. Nonetheless, the As···As arsenic bonds between the layers of the arsenic crystal in Figure 20a have the characteristics of Type-III bonding topology. They are the result of attraction between two positive sites of unequal charge density; the *V_S,min_* and *V_S,max_* values on covalently bonded As in a monolayer attract the *V_S,max_* and *V_S,min_* values of the same As atom in the interacting monolayer, respectively, explaining why the interaction between them is far from being linear (see Figure 20b, ∠As–As···As = 164.9°).

## 6. Discussion and Conclusions

This overview has highlighted several solid-state structures and chemical systems that have been known for some time, in which the role of As-centered pnictogen bonding has largely been overlooked. The underlying reason for this is that the arsenic bond has never been formally defined, nor has the role such bonds play in the formation of solid-state structures been carefully evaluated, either computationally or synthetically. This is in sharp contrast to the thousands of studies published on hydrogen and halogen bonding interactions in chemical systems that exhibit different behavior from system to system, since electron density donor sites responsible for these interactions vary between systems. The strength of the positive site on covalently bonded As is affected by the electron-withdrawing capability of R, the remainder of the molecule to which As is attached. We have highlighted illustrative examples where covalently bound arsenic in molecular entities has a significant ability to make arsenic-centered pnictogen bonds, thus contributing (at least in part) to the stabilization of these crystal lattices.

Arsenic was identified to be hypervalent in most of the molecular entities in the crystal systems explored and displayed at least three σ-holes on its electrostatic surface. The donating ability of the σ-holes in making noncovalent interactions depends on the geometrical architecture of the molecular entities that accommodate As, as well as the electron density donors that have the propensity to accept the As-centered pnictogen bonds. In the case of arsenic trihalide-containing crystals, arsenic displays an ability to donate three or more σ-hole bonds when in close proximity to surrounding electron density donors, even though it formally conceives only three σ-holes (for example, in the (15-crown-5)-trichloroarsenic complex). This appears to occur when there are more than three electron density donors on interacting partner molecules that surround the As center of the entity with which they interact.

We have observed that a covalently bound arsenic atom in molecular entities can enter into attractive engagement with a variety of electron density donors in partner molecules, including negative O, S, Se, Te, N, P, F, Cl, Br, I, and π-sites (viz. C in arene moieties) that have often been identified as donors in the formation of hydrogen-, halogen- and chalcogen-bonded interactions. This is one of the reasons why arsenic bonds are fundamentally similar, on the basis of a coulombic interaction, to hydrogen bonds, halogen bonds, and chalcogen bonds, and can be considered to be a sister noncovalent interaction. The identification and subsequent characterization of arsenic bonds between molecular entities in crystal lattices examined were made assuming that they are substantially longer than As-centered formal coordinate or covalent bonds found in the isolated tricoordinate molecules, for example, AsX_3_.

The appearance of arsenic bonds in the majority of crystals examined is accompanied by other primary or secondary interactions (such as hydrogen bonds, tetrel bonds, chalcogen bonds, and hydrogen bonds). They did not appear alone, suggesting that their strength, and indeed their very existence, may be reinforced by these other interactions. We found no specific instance where arsenic bonds act as the sole driving force (without the presence of secondary interactions) behind the formation of a crystal lattice. We believe that a very careful and comprehensive investigation of the available crystallographic databases may well lead to the identification of other electron density donors for arsenic bonds, such as a variety of π-donors that were not explicitly examined in this overview.

We found that covalently bound As in molecular entities of crystalline materials not only participates in intermolecular interactions with negative sites but is also capable of forming intramolecular interactions. The interaction distance associated with both interaction types is usually shorter, but sometimes marginally longer, than the sum of the van der Waals radii of the interacting atomic basins. This emphasizes that the concept of “less than the sum of the vdW radii” should be treated with circumspection and not as a stringent criterion for identifying a noncovalent interaction because of the inherent uncertainty in the vdW radii of atoms, given the widespread anisotropy of electron density of atoms in molecules.

Appropriate geometric criteria, based upon, but not limited to, the sum of the van der Waals radii of As and species D with which it interacts were chosen when exploring the crystallographic databases. The results show that the mode of interaction between As and D varies greatly from system to system. In many crystals, As was found to be intrinsically three-coordinate; being three-coordinate, it was electropositive and capable of becoming pseudo-six or eight-coordinate in the majority of the crystals examined. As the coordination number around As increases, the As···D bond distances become much longer than when As is strictly three-coordinate. The As···D interaction distance typically varies between 2.8 and 4.2 Å, with a peak distribution in the region of 3.1–3.7 Å for most D sites investigated. Systematic and careful examination of the intermolecular interaction of all types of As-centered noncovalent bonds in the crystals presented in this overview, especially those that constituted the histograms compiled from the CSD searches, is expected to prompt further studies that will explain the details of their interrelationships, including charge polarity. Depending on the size of the electron density donor, the interaction distance may range between 2.7 to 4.5 Å, and that the directionality of the majority of the arsenic bonds identified were in the range between about 145° and 175°. A handful of crystal systems have linear or near-linear arsenic bonds.

Statistical analysis of the CSD data provided a very tentative insight into the nature of the occurrence of As-centered close contacts in crystals. Although we have not performed a robust analysis of thousands of structures to suggest a very precise range of inter- and intramolecular arsenic bond distances and angles of approach, such an analysis in future studies should surely provide more insight into the detailed nature of the geometric aspects of arsenic bonds in crystals.

We have shown that the MESP model is useful in revealing the positive and negative sites on the electrostatic surface of a system, even though the strength of the pnictogen bonding could not be extracted from the model. Clearly, a wide variety of theoretical approaches, such as the quantum theory of atoms in molecules, noncovalent interaction index, natural bond orbital, and energy decomposition and vibrational analyses, together with binding energy calculations, would assist researchers in revealing the physical chemistry and chemical physics of As-centered pnictogen bonds in the various illustrative crystal structures explored in this overview and beyond. The chemical systems presented in this overview comprise only a few examples that were used to demonstrate the abundance of As-bonded systems widely scattered in the literature and to provide guidance to researchers, not only in the in-silico design of arsenic-centered materials in the future, but also to be used as model systems to theoretically delineate the characteristics of this important class of noncovalent interaction.

## Figures and Tables

**Table 1:** Comparison of MP2(full)/def2-TZVPPD level 0.001 a.u. isodensity envelope mapped potentials (*V_s,min_* and *V_s,max_*) with those of ωB97XD/def2-TZVPPD computed on the surfaces of the AsX_3_ molecules (X = F, Cl, Br, I). Values shown in kcal mol^−1^.

Local Most Extrema on the Surface of Specific Atom/Bond	AsF_3_		AsCl_3_		AsBr_3_		AsI_3_	
	MP2(Full)	ωB97XD	MP2(Full)	ωB97XD	MP2(Full)	ωB97XD	MP2(Full)	ωB97XD
*V_s,min_* On X (lateral portions)	−15.0	−15.4	−6.9	−7.5	−6.1	−6.7	−4.7	−5.4
*V_s,min_* on As (opposite to the triangular face formed by three X atoms)	18.1	19.4	12.8	14.8	11.5	13.7	9.4	10.1
*V_S,max_* (on As-X bond extension)	-	-	7.3	7.4	11.8	12.4	17.1	19.3
*V_S,max_* (on X-As bond extension)	40.3	40.7	28.7	30.3	25.0	27.0	20.4	21.9
*V_S,max_* (on the centroid of the triangular face formed by three X atoms)	3.9	4.1	1.0	1.1	1.1	1.4	1.6	1.9

## Data Availability

This research did not report any data.
